# Differential Mechanisms of Photosynthetic Acclimation to Light and Low Temperature in *Arabidopsis* and the Extremophile *Eutrema* *salsugineum*

**DOI:** 10.3390/plants6030032

**Published:** 2017-08-09

**Authors:** Nityananda Khanal, Geoffrey E. Bray, Anna Grisnich, Barbara A. Moffatt, Gordon R. Gray

**Affiliations:** 1 Department of Plant Sciences, University of Saskatchewan, Saskatoon, SK S7N 5A8, Canada; nityananda.khanal@agr.gc.ca (N.K.); anniethansen@icloud.com (A.G.); 2 Department of Biochemistry, University of Saskatchewan, Saskatoon, SK S7N 5E5, Canada; gbray@usk.edu; 3 Department of Biology, University of Waterloo, Waterloo, ON N2L 3G1, Canada; moffatt@uwaterloo.ca

**Keywords:** adaptive (phenotypic) plasticity, *Arabidopsis thaliana*, cold acclimation, *Eutrema salsugineum*, low temperature, photoinhibition, photosynthesis, photosynthetic acclimation

## Abstract

Photosynthetic organisms are able to sense energy imbalances brought about by the overexcitation of photosystem II (PSII) through the redox state of the photosynthetic electron transport chain, estimated as the chlorophyll fluorescence parameter 1-q_L_, also known as PSII excitation pressure. Plants employ a wide array of photoprotective processes that modulate photosynthesis to correct these energy imbalances. Low temperature and light are well established in their ability to modulate PSII excitation pressure. The acquisition of freezing tolerance requires growth and development a low temperature (cold acclimation) which predisposes the plant to photoinhibition. Thus, photosynthetic acclimation is essential for proper energy balancing during the cold acclimation process. *Eutrema salsugineum* (*Thellungiella salsuginea*) is an extremophile, a close relative of *Arabidopsis thaliana*, but possessing much higher constitutive levels of tolerance to abiotic stress. This comparative study aimed to characterize the photosynthetic properties of *Arabidopsis* (Columbia accession) and two accessions of *Eutrema* (Yukon and Shandong) isolated from contrasting geographical locations at cold acclimating and non-acclimating conditions. In addition, three different growth regimes were utilized that varied in temperature, photoperiod and irradiance which resulted in different levels of PSII excitation pressure. This study has shown that these accessions interact differentially to instantaneous (measuring) and long-term (acclimation) changes in PSII excitation pressure with regard to their photosynthetic behaviour. *Eutrema* accessions contained a higher amount of photosynthetic pigments, showed higher oxidation of P700 and possessed more resilient photoprotective mechanisms than that of *Arabidopsis*, perhaps through the prevention of PSI acceptor-limitation. Upon comparison of the two *Eutrema* accessions, Shandong demonstrated the greatest PSII operating efficiency (Φ_PSII_) and P700 oxidizing capacity, while Yukon showed greater growth plasticity to irradiance. Both of these *Eutrema* accessions are able to photosynthetically acclimate but do so by different mechanisms. The Shandong accessions demonstrate a stable response, favouring energy partitioning to photochemistry while the Yukon accession shows a more rapid response with partitioning to other (non-photochemical) strategies.

## 1. Introduction

Photosynthesis is a highly coordinated and environmentally sensitive metabolic process. Photoautrophs modulate the structure and function of their photosynthetic apparatus to changes in the environment to maintain cellular energy balance called photostasis. Photostasis results in the balancing of the light energy absorbed by the photosystems with the energy consumed by the metabolic sinks of the plants [1,2,3]. Imbalances in cellular energy are sensed through changes in chloroplastic excitation pressure (or PSII excitation pressure), reflected in the redox state of the photosynthetic electron transport chain and estimated as the in vivo chlorophyll fluorescence parameter 1-q_P_ or 1-q_L_. It is well established that modulation of light and/or temperature cause similar energy imbalances and thus, a change in PSII excitation pressure [2,3,4,5,6].

Cold acclimation refers to a process whereby plants acquire the ability to tolerate freezing (freezing tolerance). Exposure to low, non-freezing temperatures, for a period of days to weeks, triggers a series of alterations resulting in a complex reconfiguration of cellular processes at multiple levels of organization [7,8,9,10]. The level of freezing tolerance attained during cold acclimation is a coordinated response to environmental cues dependent on the genotype of the plant. The changes occurring in leaves during the transition to low temperature are thought to represent transient stress responses whereas leaves that develop at low temperature establish a new metabolic homeostasis that represents the true cold acclimated state [11,12]. It has also been demonstrated that photosynthesis interacts with other processes during cold acclimation involving crosstalk between photosynthetic redox, cold acclimation and sugar-signalling pathways to regulate plant acclimation to low temperatures [3,6]. The process of cold acclimation results in an interesting dilemma for the plant. The exposure of leaves to low temperature creates an energy imbalance between the capacity to harvest light energy and the consumption capacity of photosynthesis resulting in excess PSII excitation pressure [3,4,5]. This, in turn, can potentially result in generation of reactive oxygen species (ROS) which can lead to photoinhibition and photooxidative damage of photosystem II (PSII) and photosystem I (PSI) [13,14,15]. This predisposition to photoinhibition has made it necessary for cold acclimated plants to develop a wide array of both short- and long-term photoprotective strategies to deal with excessive light [16,17,18].

In response to increased excitation pressure, cold acclimated cereals increase their photosynthetic capacity by increasing the RuBP-regeneration and subsequently electron flux through the Calvin cycle [6,11,19]. This results in enhanced photochemical quenching through increased CO_2_ assimilation [5,6]. Alternative mechanisms for the utilization/dissipation of the excitation energy through other photochemical reactions which sustain the photosynthetic reduction of CO_2_ and/or O_2_ are also possible strategies [20,21]. Another major PSII photoprotective mechanism is the ΔpH- and zeaxanthin-dependent non-photochemical quenching (NPQ), which dissipates any excess energy not used in photosynthesis as heat to protect the PSII reaction centre from overexcitation [16,17,22]. The thermal deactivation of excess light energy occurring within the PSII reaction centre (reaction centre quenching) has also been proposed as an effective photoprotective mechanism in cold acclimated plants [18].

Regardless of the mechanism employed, which depends on the species and genera examined, plants that actively grow and develop at low temperatures (high excitation pressure) are characterized by an increased tolerance to photoinhibition, that is, these organisms are less susceptible to the light-dependent inhibition of photosynthesis as a consequence of the re-establishment of photostasis. The sensing of energy imbalances brought about by changes in temperature, irradiance or any environmental stress that alters the redox state of the photosynthetic electron transport chain and modulates excitation pressure appears to be a fundamental feature of various taxonomic groups of photosynthetic organisms including cyanobacteria, green algae, and herbaceous plants [5,6,23]. It has also been suggested that the concepts of photostasis and excitation pressure provide the context to explain phenotypic plasticity and photosynthetic performance associated with cold acclimation and photoacclimation [3,6].

Many studies have extensively characterized plant stress tolerance using *Arabidopsis thaliana*. However, more stress tolerant species may possess different and/or additional protective mechanisms that cannot be found in the commonly studied *Arabidopsis* accessions [24,25,26]. *Eutrema salsugineum* (*Thellungiella salsuginea*) is an alternative plant model species particularly well suited for the examination of stress tolerance as this genus is also part of the Brassicaceae family and therefore closely related to *Arabidopsis* [27]. However, *Eutrema* is often referred to as an extremophile, owing, in part, to its high capacity to withstand various abiotic stresses such as freezing, water deficit, nutrient-deficiency and salinity [28,29,30,31,32,33,34,35] Primarily, two accessions from stress-prone, geographically diverse locations have been examined; the Shandong accession originating from Shandong Province, China and the Yukon accession, native to the Yukon Territory, Canada. These two accessions grow under contrasting natural habitats with Yukon being subjected to a subarctic and semi-arid climate and Shandong growing under warm temperate regions in high-salinity coastal areas with more frequent precipitation [28,34]. Plant populations evolved in different ecological niches are known to have differential adaptive specificities of photosynthetic properties [36,37]. Therefore, we would anticipate that the Shandong and Yukon accessions would perform, at least in part, based on their ecological backgrounds.

*Eutrema* has been used as a model for the study of cold acclimation and freezing tolerance [31,38,39]. While the study of [39] demonstrated that *Eutrema* and *Arabidopsis* did not differ in their constitutive level of freezing tolerance or short-term cold acclimation capacity, *Eutrema* outperformed *Arabidopsis* in long-term acclimation capacity suggesting a wider phenotypic plasticity for the trait of freezing tolerance. Furthermore, it was demonstrated that growth conditions, specifically irradiance, were determinants of the level of freezing tolerance attained during cold acclimation suggesting a role for photosynthetic processes in adaptive stress responses. Another study examining the cold-induced proteome showed nearly half of the identified cold-responsive proteins were associated with various aspects of chloroplast metabolic processes [40]. However, the role of photosynthetic acclimation in this process remains unknown for *Eutrema*, despite the fact transcript profiling and stress induced gene expression studies are almost always enriched by the differential expression of photosynthesis-related genes [29,30,33,34,41,42,43,44]. Previous studies have focused on the role of photosynthesis under salinity stress and photoinhibitory responses [45,46,47].

This study reports the results of comparative experiments aimed to characterize basic photosynthetic properties of the Yukon and Shandong accessions of *Eutrema* as well as *Arabidopsis* under non-acclimating and cold acclimated conditions. Furthermore, at both non-acclimating and cold acclimating conditions, three different growth regimes were examined which varied in day/night temperatures, photoperiod and irradiance. In addition to an examination of photosynthetic pigments, chlorophyll *a* fluorometry and photosystem I (PSI) spectroscopy were utilized to examine photosynthetic parameters with an underlying hypothesis that Yukon, Shandong and *Arabidopsis* possess differential capacities to modulate photosynthetic responses to light and temperature based on their contrasting ecophysiological backgrounds.

## 2. Results

### 2.1. Growth Regimes

It is well established that changes in light and temperature are sensed by the redox state of the photosynthetic electron transport chain and reflected in PSII excitation pressure [3,4,5]. Thus, different growth conditions will result in differential PSII excitation pressures. By manipulating growth parameters one can also manipulate PSII excitation pressure. Three different growth regimes were utilized in this study which varied in day/night temperatures, photoperiod and irradiance (Table 1). In addition, both non-acclimating and cold acclimating conditions were examined (Table 1). These conditions were chosen so as to be representative of growth conditions typically used to propagate *Eutrema* and *Arabidopsis* plant material and are referred to as the Yukon, Shandong or *Arabidopsis* growth regimes (see [24] and references contained within).

The daily average temperature (DAT) varied by 1.0 and 0.2 °C at non-acclimating and cold acclimating growth conditions respectively (Table 1). In contrast, the daily photon receipt (DPR) was highest in the Yukon, followed by the Shandong and finally the *Arabidopsis* growth regimes (Table 1). The Yukon and Shandong growth regimes exhibited a 3.3- and 2.5-fold increase in DPR in comparison to the *Arabidopsis* growth regime. In addition, the Yukon growth regime showed a 1.3-fold increase in DAT compared to the Shandong growth regime (Table 1). In this study it is clear that DPR plays a much more prominent role in contributing to PSII excitation pressure under the various growth regimes. The use of multiple growth regimes was not to mimic natural conditions, but rather to demonstrate increased PSII excitation pressure (1-q_L_) with different combinations of growth parameters (irradiance, temperature, photoperiod) that are reflective of a wide variety of controlled environment studies using *Arabidopsis* or *Eutrema*.

### 2.2. Photosynthetic Pigmentation

Growth conditions affect variables such as leaf area and thickness that in turn has an effect on the leaf pigmentation. Moreover, leaf pigment composition can also reflect the acclimation and adaptation strategies of plants in response to various environmental factors that affect photosynthesis.

#### 2.2.1. Analysis of Chlorophyll and Carotenoid Contents

Combined analysis showed a significant difference in chlorophyll content per unit leaf fresh weight (ChlFW) between the accessions (*P* < 0.001), between the growth regimes (*P* = 0.037) and between the cold acclimation status (*P* < 0.001) of the plants (Table 2). For this parameter, significant two or three-way interactions were found between the genotypic and environmental factors: accessions by growth regime (*P* = 0.014), accessions by acclimation (*P* < 0.001), growth regime by cold acclimation (*P* = 0.04) and accession by growth regime by cold acclimation (*P* = 0.005). Chlorophyll content per unit leaf area (ChlLA) also varied significantly between the accessions (*P* < 0.001), across the growth regimes (*P* < 0.001) and cold acclimation status (*P* = 0.036) along with a significant two and three way interactions between accessions, growth regime and acclimation status (*P* < 0.001 to 0.05; Table 2).

Similarly, carotenoid content per unit leaf weight (CarFW) differed significantly between the accessions (*P* < 0.001) and between the acclimation status (*P* < 0.001). However, no significant difference was observed for this parameter across the growth regimes (*P* = 0.188; Table 2). CarFW was affected significantly by the interactions between the accessions and growth regime (*P* = 0.004), between accessions and acclimation status (*P* < 0.001), between growth regime and cold acclimation (*P* = 0.008) and between accession, growth regime and acclimation status (*P* = 0.039). Carotenoid content per unit leaf area (CarLA) also displayed highly significant difference between the accessions, growth regimes and acclimation status (*P* < 0.001 for all factors; Table 2) with significant two and three way interactions between the genotypic and environmental factors (*P* < 0.001 to 0.015).

In the overall analysis, the accessions did not vary significantly in the chlorophyll *a*:*b* ratio (Chl *a*:*b*; *P* = 0.91), but significant alterations in the values were observed across the growth regimes (*P* < 0.001) and acclimation status (*P* = 0.004). For this parameter, significant interactions were found between accessions and growth regime (*P* < 0.001), between accession and acclimation status (*P* < 0.001) and between accession, growth regime and acclimation status (*P* = 0.002). However, no significant interaction was observed between growth regime and acclimation status (*P* = 0.099) in Chl *a*:*b* (Table 2). Unlike other pigmentation parameters, the chlorophyll:carotenoid ratio (Chl:Car) did not differ significantly between the accessions (*P* = 0.389) and across growth regimes *(P* = 0.06). However, acclimation status altered the ratio significantly (*P* < 0.001), with significant interactions between the accession and acclimation (*P* = 0.018), between accessions and growth regimes (*P* = 0.019), and between accessions, growth regimes and acclimation status (*P* = 0.001; Table 2). The significant interactions between the accessions with growth regimes and measurement temperatures imply that the accessions adopt differential photosynthetic adjustment strategies in response to changes in growth regimes and measurement conditions.

#### 2.2.2. Effect of Cold Acclimation on Pigmentation across Growth Regimes

Cold acclimation had differential effects on photosynthetic pigmentation between the accessions. Upon cold acclimation for 3 weeks, Yukon plants showed a significant decrease in ChlFW under the Yukon and Shandong growth regimes, but there was no significant change in this parameter in the *Arabidopsis* growth regime. Shandong plants consistently displayed a significant decrease in ChlFW upon cold acclimation across all three growth regimes. On the other hand, *Arabidopsis* plants showed no significant change in ChlFW upon cold acclimation under all three growth regimes. The ChlLA remained more or less stable without any significant change due to cold acclimation in all three accessions across growth regimes (Table 2).

Yukon and *Arabidopsis* plants showed similar trends in CarFW due to cold acclimation, with significant increases in CarFW in the Yukon and Shandong growth regimes, while having no significant change in *Arabidopsis* growth regime. Contrarily, Shandong plants did not show significant changes in CarFW upon cold acclimation across all regimes (Table 2).

The accessions did not show any definite trends in Chl *a*:*b* upon cold acclimation. Yukon plants showed a significant increase in Chl *a*:*b* in the Yukon and Shandong growth regimes while having no significant change in the *Arabidopsis* growth regime. Contrarily, upon cold acclimation, Shandong plants underwent a significant increase in Chl *a*:*b* in the Shandong growth regime, while displaying no significant change in this parameter in the Yukon growth regime and a significant decrease in the *Arabidopsis* growth regime. On the other hand, cold acclimation brought about no significant change in Chl *a*:*b* in *Arabidopsis* plants in the Yukon and *Arabidopsis* growth regimes and surprisingly decreased the value of this parameter in the Shandong growth regime. A generalized observation of cold acclimation was a decrease in the Chl:Car ratio in all accessions across all three growth regimes. However, in some of the cases, the change in the ratio was not statically significant (Table 2).

With only a few exceptions, the *Eutrema* accessions showed a significantly higher content of both chlorophyll and carotenoid pigments both on a per unit fresh weight and per unit leaf area basis across all growth regimes under both acclimated and non-acclimated conditions. Again with few exceptions, Yukon plants seemed to be significantly higher than or at par with Shandong plants for the pigment content considered. Except for the *Arabidopsis* growth regime, Shandong plant had either a significantly higher or similar Chl *a*:*b* ratio across all regimes, while the relationship was opposite in the *Arabidopsis* growth regime. Contrarily, for the Chl:Car ratio, Shandong plants appeared to be significantly lower than or equivalent to the Yukon and *Arabidopsis* plants across all growth regimes. In most cases, *Arabidopsis* and Yukon plants displayed similar levels of the Chl:Car ratio across all growth regimes (Table 2). Some exceptions in the relative content of the photosynthetic pigment parameters suggest that there was an interaction between the accessions and the growth regimes. The above results show that photosynthetic pigmentation parameters differed across growth regimes with significant interactions between the accessions and the growth regimes. However, due to the multifactor variation in the growth regimes including the temperature, irradiance and photoperiod, the results did not differentiate the effects of individual factors on the pigmentation parameters.

### 2.3. Comparative PSII Photochemistry

**Figure 1:**
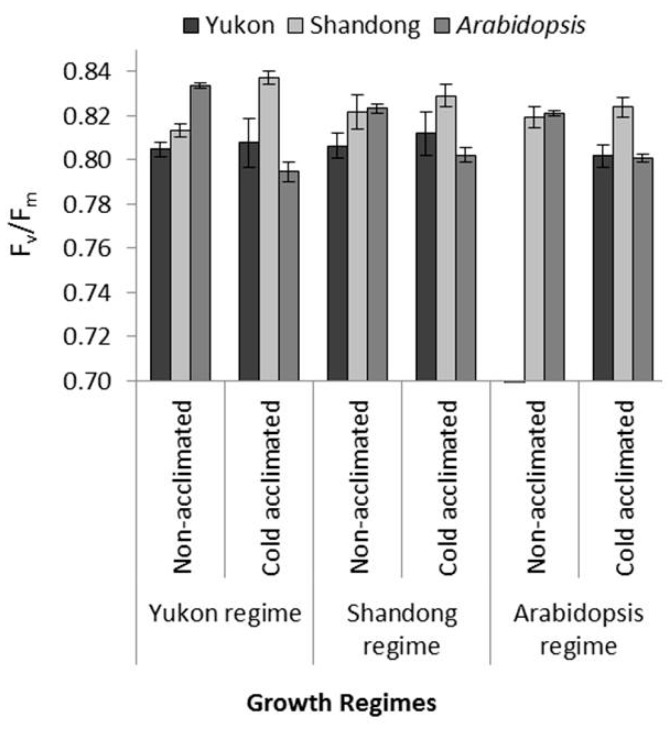
Maximum quantum efficiency of PSII (F_v_/F_m_) of non-acclimated and cold acclimated *Eutrema* accessions and *Arabidopsis* developed under three different growth regimes. Yukon accession (■); Shandong accession (■); *Arabidopsis* (■). No results were obtained for the Yukon accession in the non-acclimated *Arabidopsis* regime due to poor growth. Values represent means ± SE (*n* = 3 to 6). PSII = photosystem II; SE = standard error. Means were grouped by Fisher’s individual error rate at significance level of 0.05.

A comparison of the maximum quantum efficiencies of PSII (F_v_/F_m_) suggests that growth regime and acclimation status had minor but differential effects on Yukon, Shandong and *Arabidopsis* plants (Figure 1). The accessions differed significantly in F_v_/F_m_ values (*P* < 0.0001). The acclimation status had significant effects on F_v_/F_m_ (*P* < 0.0001), while the growth regimes per se had no significant effects *(P* = 0.64). Significant two-way (accessions by acclimation, accessions by growth regime, and acclimation by growth regime) and three-way interactions (accessions by growth regime by acclimation status) *(P* < 0.0001) suggest a differential responses of accessions to the environmental conditions. The *Eutrema* accessions displayed a more consistent F_v_/F_m_ trend than that of *Arabidopsis* across the growth regimes and acclimating conditions. However, differences in *Eutrema* accessions were evident by consistently higher F_v_/F_m_ values of Shandong (0.81 to 0.84) than those of Yukon (0.79 to 0.82) (Figure 1). Unlike *Eutrema*, *Arabidopsis* plants underwent a consistent decrease in F_v_/F_m_ values upon cold acclimation across all growth regimes. The cold-acclimated values of *Arabidopsis* (0.77 to 0.80) remained lower than the non-acclimated control values (0.82 to 0.83) (Figure 1). Under non-acclimated conditions, the F_v_/F_m_ values of *Arabidopsis* were comparable to those of Shandong plants (Figure 1).

### 2.4. Photoinhibition and Recovery of PSII

**Figure 2:**
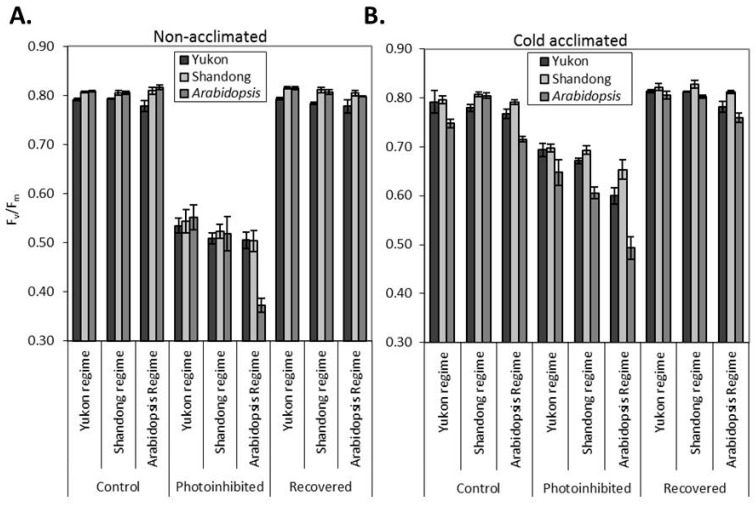
Photoinhibition and recovery measured as changes in maximum quantum efficiency of PSII (F_v_/F_m_) for (**A**) non-acclimated and (**B**) cold acclimated *Eutrema* accessions and *Arabidopsis* developed under three different growth regimes. Yukon accession (■); Shandong accession (■); *Arabidopsis* (■). Photoinhibition occurred for 4 h with a photosynthetic photon flux density (PPFD) of 1750 μmol photons m^−2^ s^−1^ at 7 °C. Recovery occurred at 22 °C under dim light (30 μmol photons m^−2^ s^−1^) for 24 h. Values represent means ± SE (*n* = 3 to 6). PSII = photosystem II; SE = standard error. Fisher’s individual error rate at significance level of 0.05 was used for inter-specific means comparison.

Exposure of non-acclimated and cold acclimated *Arabidopsis* and *Eutrema* plants to a high irradiance of 1750 μmol photons m^−2^ s^−1^ coupled with low temperature (7 °C) for 4 h resulted in differential levels of photoinhibition between the accessions across the growth regimes (Figure 2). Cold acclimation significantly enhanced the tolerance to photoinhibition. The reduction in F_v_/F_m_ was significantly (*P* < 0.001) higher (32 to 54%) in non-acclimated plants than that of cold acclimated plants (12 to 31%; Figure 2). Under non-acclimating conditions, plants grown with an irradiance of 250 μmol photons m^−2^ s^−1^ exhibited a reduction of F_v_/F_m_ in the range of 32 to 36%, while those values for plants grown with an irradiance of 100 μmol photons m^−2^ s^−1^ were 35 to 54%. The cold acclimated plants from a growth irradiance of 250 μmol photons m^−2^ s^−1^ had a lower extent of photoinhibition (12 to 25%) than those from the lower growth irradiance (17 to 31%; Figure 2). The interactions of experimental accessions with growth regimes and acclimation status resulted in variation of the extent of photoinhibition between the accessions. Yukon and Shandong plants displayed relatively more stable values of F_v_/F_m_ with a lower extent of photoinhibition than *Arabidopsis* (Figure 2). Under higher growth irradiance (250 μmol photons m^−2^ s^−1^), photoinhibitory responses of *Arabidopsis* plants were at par with *Eutrema*. However, when grown under lower irradiance (100 μmol photons m^−2^ s^−1^), *Arabidopsis* plants showed greater susceptibility to photoinhibition than the *Eutrema* accessions. For instance, with a growth irradiance of 250 μmol photons m^−2^ s^−1^, the non-acclimated plants of all three experimental accessions underwent photoinhibition by 32 to 36% and the cold acclimated plants by 12 to 24%. On the other hand, *Arabidopsis* underwent photoinhibition by 54% and 31% in non-acclimated and cold-acclimated plants respectively, while the corresponding values for *Eutrema* were 35 to 38% in non-acclimated plants and 17 to 22% in cold acclimated plants under lower growth irradiance (100 μmol photons m^−2^ s^−1^ at *Arabidopsis* growth regime). After the release of photoinhibitory treatments, plants were kept at room temperature (22 °C) with a low irradiance of approximately 30 μmol photons m^−2^ s^−1^. After 24 h of releasing the photoinhibitory stress, all plants fully recovered their F_v_/F_m_ to the equivalent level of the control plants (Figure 2). This recovery suggests that the photoinhibition in all accessions was reversible, suggesting no differences in the effectiveness of repair of the photosynthetic apparatus or permanent photooxidative damage.

### 2.5. Chlorophyll Fluorescence Quenching Analyses

#### 2.5.1. Excitation Pressure (1-q_L_)

**Figure 3:**
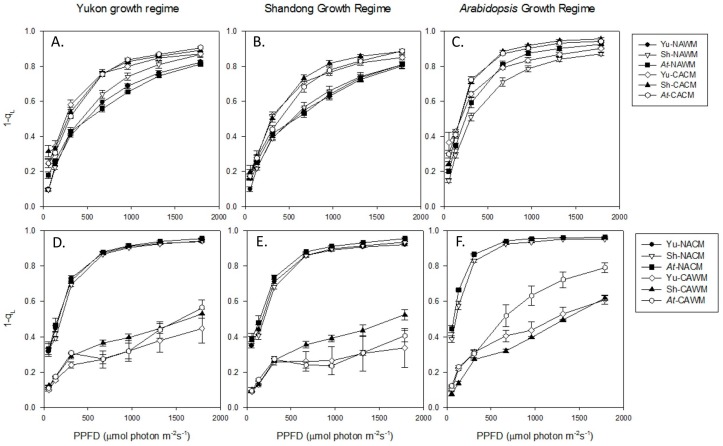
Light response curves of 1-q_L_ for *Eutrema* accessions and *Arabidopsis* developed under three different growth regimes. Non-acclimated and cold acclimated plants of Yukon (Yu), Shandong (Sh) and *Arabidopsis* (*At*) were subjected to their respective growth temperatures (**A**–**C**) as well as reciprocal temperature measurements (**D**–**F**). No results were obtained for the Yukon accession in the non-acclimated *Arabidopsis* regime due to poor growth. Values represent means ± SE (*n* = 3 to 6). CACM = cold acclimated cold-measured; CAWM = cold acclimated warm-measured; NACM = non-acclimated cold measured; NAWM = non-acclimated warm-measured; PSII = photosystem II; 1-q_L_ = PSII excitation pressure; SE = standard error.

The parameter 1-q_L_ reflects the redox poise of the primary quinone electron acceptor of PSII (Q_A_) and is an estimate of PSII excitation pressure. In all accessions under various growth regimes, 1-q_L_ increased non-linearly with the increase in measuring photosynthetic photon flux densities (PPFDs) (Figure 3). With respect to measurement at respective growth temperatures, the 1-q_L_ light response curves of non-acclimated and cold acclimated plants clustered distinctly, displaying higher 1-q_L_ in cold acclimated plants (Figure 3A–C). This indicates that the temperature-dependence of 1-q_L_ is not fully compensated through cold acclimation. On the other hand, measurement of non-acclimated and cold acclimated plants at reciprocal temperatures across the range of PPFDs resulted in contrasting interactions of measuring irradiance and temperature (Figure 3D–F). With reference to the control (non-acclimated warm measured; NAWM), the non-acclimated cold measured (NACM; cold shock) resulted in the acceleration of 1-q_L_, while the cold acclimated warm measured (CAWM) gave rise to a relaxation of 1-q_L_.

Similarly, the 1-q_L_ trend was also affected by the growth conditions. This is substantiated by the fact that plants grown in *Arabidopsis* growth regime displayed a higher 1-q_L_ than those in the Yukon and Shandong growth regimes across the range of measuring PPFDs under different measuring temperatures and acclimation status (Figure 3). Similarly, in response to increasing PPFDs, the plants grown in Shandong growth regime showed a slightly lower 1-q_L_ than those in Yukon growth regime. The taxonomic differences in 1-q_L_ were negligible under all experimental conditions, except for those grown in *Arabidopsis* growth regime (Figure 3). The measurement of non-acclimated plants at low temperature (NACM) triggered an acceleration of 1-q_L_ that was substantially lower in Shandong (42%) than that of *Arabidopsis* and Yukon plants that had identical values of 51% (Table 3). Similarly, cold acclimation increased relaxation in 1-q_L_, and was higher in plants of *Arabidopsis* (20%) followed by Yukon (17%) and then Shandong (13%) (Table 3). When cold acclimated plants were exposed to non-acclimated (control) growth temperature (CAWM), there was substantial relaxation in 1-q_L_ and that was estimated to be 58%, 48% and 52% on the average for Yukon, Shandong and *Arabidopsis* plants respectively (Table 3).

#### 2.5.2. Relative Electron Transport Rate (RETR_PSII_)

**Figure 4:**
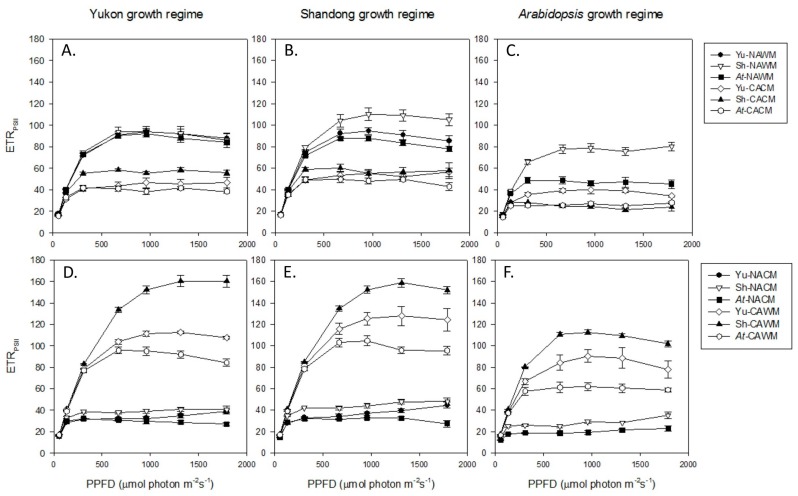
Light response curves of RETR_PSII_ for *Eutrema* accessions and *Arabidopsis* developed under three different growth regimes. Non-acclimated and cold acclimated plants of Yukon (Yu), Shandong (Sh) and *Arabidopsis* (*At*) subjected to their respective growth temperatures (**A**–**C**) as well as reciprocal temperature measurements (**D**–**F**). No results were obtained for the Yukon accession in the non-acclimated *Arabidopsis* regime due to poor growth. Values represent means ± SE (*n* = 3 to 6). CACM = cold acclimated cold measured; CAWM = cold acclimated warm-measured; RETR_PSII_, non-cyclic electron transport rate through PSII; NACM = non-acclimated cold measured; NAWM = non-acclimated warm-measured; PSII = photosystem II; SE = standard error.

Yukon, Shandong and *Arabidopsis* plants exhibited comparable effects of measurement temperature, cold acclimation and growth regimes on the light response curves of RETR_PSII_ (Figure 4). Under the respective growth temperatures, cold acclimated plants showed less RETR_PSII_ in the range of experimental PPFDs along with light-saturation of RETR_PSII_ at lower PPFDs, compared to that of non-acclimated plants (Figure 4A–C). Measurement of non-acclimated plants at low-temperature (NACM) displayed a further depression of RETR_PSII_ as the indication of an inhibitory effect of low temperature on photosynthesis of non-acclimated plants (Figure 4D–F). On the other hand, measurement of cold acclimated plants at warm temperature (CAWM) augmented the RETR_PSII_ that substantially exceeded the control values of RETR_PSII_ from NAWM plants. Similarly, compared to the RETR_PSII_ of NACM, the cold acclimated cold measured (CACM) values were higher in the light response curves. While the experimental accessions displayed common trends across the various growth regimes, quantitative differences between their responses were also evident. Shandong plants generally outperformed both of its counterparts in that *Arabidopsis* remained mostly at the lower scale, while Yukon was in an intermediate position between the Shandong and *Arabidopsis* plants in the light response curves. However, there are a few exceptions to this generalization indicating differential interactions between the genotype and environmental conditions (Figure 4). The NACM depression of RETR_PSII_ was highest in *Arabidopsis* plants and lowest in Shandong plants across all growth regimes (Table 3). These amounted to 49% in Shandong, 54% in Yukon and 57% in *Arabidopsis* plants. On the other hand, the increases in RETR_PSII_ due to cold acclimation were higher in *Arabidopsis* (40%) followed by Yukon (34%) and then Shandong (32%). When cold acclimated plants were exposed to non-acclimating (control) growth temperatures (CAWM), there was a rapid escalation of RETR_PSII_ that was estimated to be 118%, 105% and 94%, for Shandong, Yukon and *Arabidopsis* plants respectively (Table 3).

#### 2.5.3. Basal Fluorescence Quenching (q_O_)

**Figure 5:**
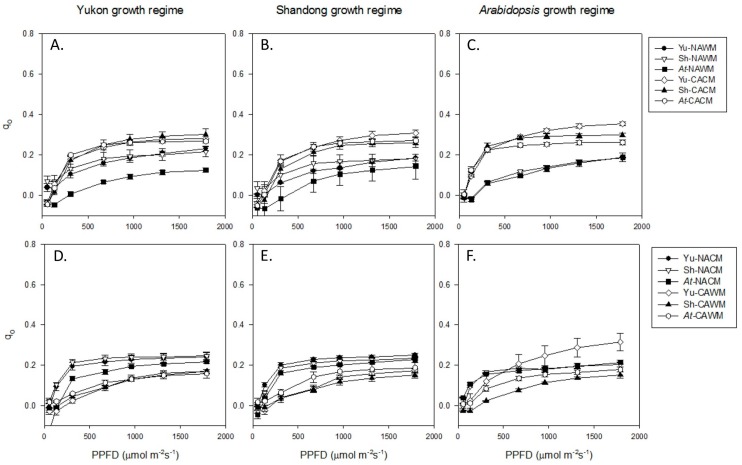
Light response curves of q_O_ for *Eutrema* accessions and *Arabidopsis* developed under three different growth regimes. Non-acclimated and cold acclimated plants of Yukon (Yu), Shandong (Sh) and *Arabidopsis* (*At*) subjected to their respective growth temperatures (**A**–**C**) as well as reciprocal temperature measurements (**D**–**F**). No results were obtained for the Yukon accession in the non-acclimated *Arabidopsis* regime due to poor growth. Values represent means ± SE (*n* = 3 to 6). CACM = cold acclimated cold measured; CAWM = cold acclimated warm-measured; NACM = non-acclimated cold measured; NAWM = non-acclimated warm-measured; q_O_ = coefficient of basal fluorescence quenching; SE = standard error.

The coefficient q_O_ is an indicator of dissipation of excitation energy from light-harvesting antenna of PSII (antenna quenching). Excluding the results of the *Arabidopsis* growth regime where a complete set of comparative data were lacking, cold acclimated plants had consistently higher values of q_O_ than non-acclimated plants when measured at their respective growth temperatures (Figure 5A–C). The results of reciprocal measurement temperatures were virtually opposite from those of growth temperatures where light response curves of non-acclimated plants remained consistently above those of cold acclimated plants (Figure 5D–F). All experimental accessions exhibited similar responses of q_O_ upon cold acclimation. On the other hand, under non-acclimated conditions the q_O_ of *Arabidopsis* plants remained quantitatively lower than *Eutrema* accessions (Figure 5A–C). The experimental accessions exhibited differential effects of cold acclimation and measurement temperature on q_O_. These responses were well discernible at PPFDs higher than 310 μmol photons m^−2^ s^−1^ (Figure 5). Low measuring temperature of the non-acclimated plants (NACM) triggered a rise in q_O_ which was calculated for the irradiance levels higher than 310 μmol photons m^−2^ s^−1^. This rise in q_O_ was significantly higher in *Arabidopsis* plants (99% on the average) followed distantly by Yukon (39%) and Shandong (26%) (Table 3). Similarly, cold acclimation resulted in a gain in q_O_ with increases in PPFDs beyond 310 μmol photons m^−2^ s^−1^. This increase was significantly higher in *Arabidopsis* plants (30% on the average) followed by Yukon (16%) and Shandong (13%). When cold acclimated plants were exposed to non-acclimating (control) growth temperatures (CAWM) there was substantial down-shift in the light response curve of q_O_. Such a subsidence in q_O_ due to warm measuring temperature was estimated for the PPFD levels above 310 μmol photons m^−2^ s^−1^. This was higher in plants of *Eutrema* (50% in Yukon and 52% in Shandong) than that in *Arabidopsis* (41%) (Table 3). These results show that the effects of low measuring temperature and cold acclimation on q_O_ was more pronounced in *Arabidopsis* plants than in *Eutrema*.

#### 2.5.4. Excitation Energy Partitioning with Increasing Irradiance

**Figure 6:**
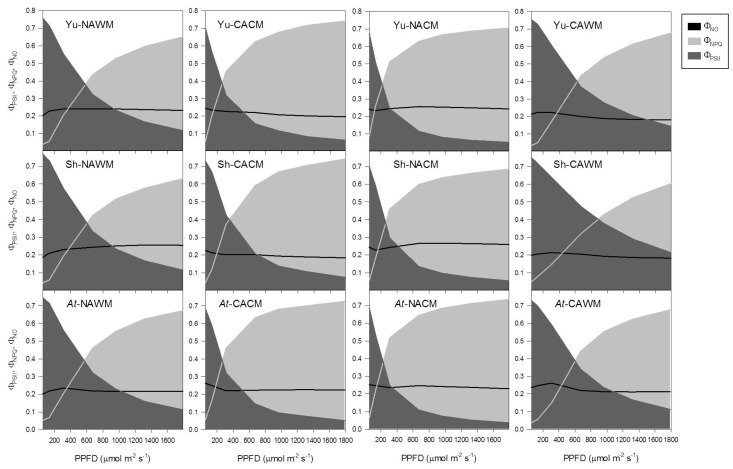
Partitioning of excitation energy as a function of irradiance for *Eutrema* accessions and *Arabidopsis*. The fraction of excitation energy flow via PSII photochemistry (Φ_PSII_) and non-photochemical dissipation pathways (Φ_NO_ and Φ_NPQ_) in Yukon (Yu), Shandong (Sh) and *Arabidopsis* (*At*) were estimated for plants developed under a Yukon growth regime. Non-acclimated and cold acclimated plants were subjected to their respective growth temperatures as well as reciprocal temperature measurements. CACM = cold acclimated cold measured; CAWM = cold acclimated warm-measured; NACM = non-acclimated cold measured; NAWM = non-acclimated warm-measured; NPQ = non-photochemical quenching; PSII = photosystem II; Φ_NO_ = efficiency of constitutive non-photochemical energy dissipation and fluorescence; Φ_NPQ_ = efficiency of light dependent NPQ; Φ_PSII_ = PSII operating efficiency.

**Figure 7:**
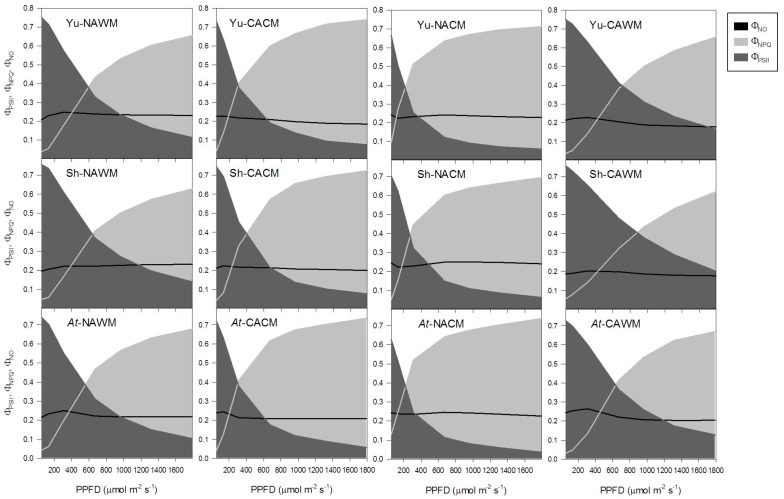
Partitioning of excitation energy as a function of irradiance for *Eutrema* accessions and *Arabidopsis*. The fraction of excitation energy flow via PSII photochemistry (Φ_PSII_) and non-photochemical dissipation pathways (Φ_NO_ and Φ_NPQ_) in Yukon (Yu), Shandong (Sh) and *Arabidopsis* (*At*) were estimated for plants developed under a Shandong growth regime. Non-acclimated and cold acclimated plants were subjected to their respective growth temperatures as well as reciprocal temperature measurements. CACM = cold acclimated cold measured; CAWM = cold acclimated warm-measured; NACM = non-acclimated cold measured; NAWM = non-acclimated warm-measured; NPQ = non-photochemical quenching; PSII = photosystem II; Φ_NO_ = efficiency of constitutive non-photochemical energy dissipation and fluorescence; Φ_NPQ_ = efficiency of light dependent NPQ; Φ_PSII_ = PSII operating efficiency.

**Figure 8:**
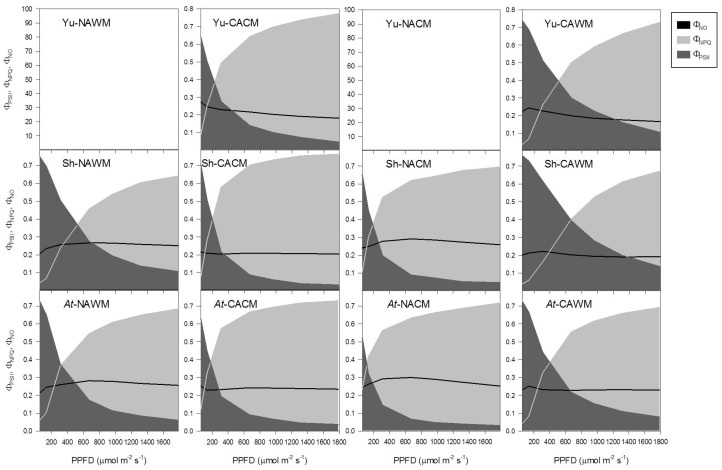
Partitioning of excitation energy as a function of irradiance for *Eutrema* accessions and *Arabidopsis*. The fraction of excitation energy flow via PSII photochemistry (Φ_PSII_) and non-photochemical dissipation pathways (Φ_NO_ and Φ_NPQ_) in Yukon (Yu), Shandong (Sh) and *Arabidopsis* (*At*) were estimated for plants developed under an *Arabidopsis* growth regime. Non-acclimated and cold acclimated plants were subjected to their respective growth temperatures as well as reciprocal temperature measurements. The blank graphs of the Yukon accession denote no results obtained due to poor growth of the accession in that condition. CACM = cold acclimated cold measured; CAWM = cold acclimated warm-measured; NACM = non-acclimated cold measured; NAWM = non-acclimated warm-measured; NPQ = non-photochemical quenching; PSII = photosystem II; Φ_NO_ = efficiency of constitutive non-photochemical energy dissipation and fluorescence; Φ_NPQ_ = efficiency of light dependent NPQ; Φ_PSII_ = PSII operating efficiency.

Increasing measurement PPFD resulted in the non-linear decrease in the efficiency of PSII photochemistry (Φ_PSII_) with the concomitant increase in the non-photochemical dissipation (NPQ) of the excitation energy in all growth regimes and measurement temperatures (Figure 6, Figure 7 and Figure 8). The fraction of dissipated energy was discerned into two components: the first component being the light independent, constitutive non-photochemical energy dissipation and fluorescence (Φ_NO_), and the second component being the light regulated, predominantly ΔpH- and/or zeaxanthin-dependent non-photochemical dissipation within the PSII antenna (Φ_NPQ_). The Φ_NO_ exhibited a negligible effect of experimental accessions, growth regimes and measurement temperature, remaining more or less constant (approximately 0.2). It was the Φ_NPQ_ that competed with Φ_PSII_ in the excitation energy partitioning in response to changes in the measurement temperature, measurement irradiance and the growth regimes (Figure 6, Figure 7 and Figure 8).

The acclimation status of plants and measurement temperature triggered a marked effect, while the growth regimes and plant accessions had only subtle effects on the partitioning of the excitation energy. When measured at respective growth temperatures, cold acclimated plants responded to increasing PPFD with a more rapid down-regulation of Φ_PSII_ with a proportionate increase in Φ_NPQ_ (Figure 6, Figure 7 and Figure 8). Generally, the NAWM plants displayed higher Φ_PSII_ than Φ_NPQ_ depending on the measurement PPFD and accessions from the three different growth regimes. In contrast, the CACM plants showed more competitive Φ_NPQ_ that surpassed Φ_PSII_ depending on the growth regimes and accessions (Figure 6, Figure 7 and Figure 8). Measurement at reciprocal temperatures showed a more contrasting trend of energy partitioning in non-acclimated and cold acclimated plants (Figure 6, Figure 7 and Figure 8). The Φ_PSII_ was surpassed by Φ_NPQ_ at much lower PPFDs in NACM plants compared to the CAWM counterparts.

The effect of growth regimes on energy partitioning was also evident. In general, at given PPFD levels and experimental conditions the *Arabidopsis* growth regime had lower Φ_PSII_ and higher Φ_NPQ_ than the Yukon and Shandong growth regimes (Figure 6, Figure 7 and Figure 8).

#### 2.5.5. Excitation Energy Partitioning with Increasing Excitation Pressure

It is intriguing to examine whether the key photosynthetic correlates of *Eutrema* accessions and *Arabidopsis* respond similarly to excitation pressure. Separate regression results of Shandong, Yukon and *Arabidopsis* plants display that changes in excitation pressure explain the variation in photochemical and non-photochemical correlates of photosynthesis (Figure S1). Though the trends of all experimental accessions were fairly similar, some differences in slopes were distinguishable. In general, Shandong is quantitatively more responsive to excitation pressure for Φ_PSII_ and Φ_NPQ_ and *Arabidopsis* for q_O_ (Figure S1).

The high correlation of excitation pressure with photochemical and NPQ parameters led to the consideration that excitation pressure may serve as a unifying determinant of energy partitioning. To examine the pattern of energy partitioning in response to excitation pressure, the results of energy fractionation from different growth regimes and measurement conditions were plotted against the excitation pressure (Figures S2–S4). When measured at growth irradiance, *Arabidopsis* plants displayed relatively lower Φ_PSII_ or higher Φ_NPQ_ at a given excitation pressure than did *Eutrema* accessions. At the reciprocal measurement temperatures, cold acclimated plants displayed higher sensitivity of energy partitioning pattern to the changes in excitation pressure than the non-acclimated plants (Figures S2–S4).

### 2.6. Redox State of PSI and the Intersystem Electron Pool

Exposure of leaves to far-red (FR) light results in an absorbance change at 820 nm (Δ*A*_820_), an indicator of the oxidation of P700. The P700^+^ is transiently reduced with the application of saturating single-turnover (ST) flash or multiple turnover (MT) flashes in the presence of background FR light. The ratio of the extent of reduction of P700 triggered by the MT flash to the ST flash is an indicator of the number of electrons stored in the intersystem electron transport chain (e^−^/P700) or intersystem electron pool size.

#### 2.6.1. PSI Oxidation

**Figure 9:**
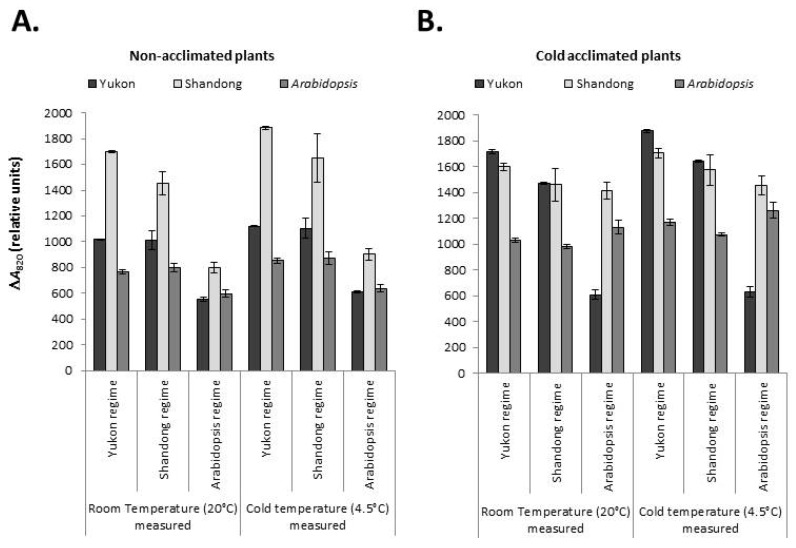
P700 oxidation measured as Δ*A*_820_ for (**A**) non-acclimated and (**B**) cold acclimated *Eutrema* accessions and *Arabidopsis* developed under three different growth regimes. Yukon accession (■); Shandong accession (■); *Arabidopsis* (■). Plants were measured at room (20 °C) and cold (4.5 °C) temperatures. Values represent means ± SE (*n* = 4 to 9). SE = standard error.

Yukon, Shandong and *Arabidopsis* plants differed significantly (*P* < 0.001) for P700 oxidation within and across the growth regimes, acclimation status and measurement temperatures. The accessions also displayed significant interactions (*P* < 0.001) with growth regime and cold acclimation for the extent of P700 oxidation (Figure 9). In all experimental accessions, the effect of measurement temperature on P700 oxidation was consistently similar. The generalized effect was that all three experimental accessions showed a consistently higher extent of P700 oxidation at cold temperature measurement than at warm temperature measurement (Figure 9). The differences between the corresponding values were significant, though the magnitudes of differences were not very high (2.9% to 13.78% on average). Unlike the low temperature measurement, the effect of cold acclimation on P700 oxidation was variable between the experimental accessions (Figure 9). Cold acclimation enhanced the capacity of P700 oxidation in Yukon (3% to 68% increase) and *Arabidopsis* plants (22% to 98% increase) grown across all growth regimes. Shandong plants grown at 100 μmol photons m^−2^ s^−1^ (*Arabidopsis* growth regime) also displayed a significant increase (61% to 77%) in P700 oxidation due to cold acclimation. However, when Shandong plants were grown under a growth irradiance of 250 μmol photons m^−2^ s^−1^ (Shandong and Yukon growth regimes) the modulating effect of cold acclimation on P700 oxidation disappeared (−9.5% to 0.8% change).

In all experimental accessions, an increase in growth irradiance from 100 (*Arabidopsis* growth regime) to 250 μmol photons m^−2^ s^−1^ (Yukon and Shandong growth regimes) significantly increased P700 oxidation under non-acclimated conditions (Figure 9). However, Shandong and Yukon plants had a better response to growth irradiance in relation to P700 oxidation with a greater magnitude of increase (82% to 112% increases) than that of *Arabidopsis* (28% to 37% increases) under non-acclimated conditions. Similarly, the cold acclimated Yukon accession distinguished itself with the highest response to growth irradiance with a 142% to 197% increase in P700 oxidation due to increasing growth irradiance. The Shandong accession followed the trend with a moderate response (4% to 17% increases) in the amount of oxidized P700 with the increase in irradiance. However, cold acclimated *Arabidopsis* plants showed contrasting responses in that the P700 oxidation was 7% to 15% lower in plants grown under higher growth irradiances. Non-acclimated Shandong plants consistently displayed higher amounts of oxidized P700 than both Yukon and *Arabidopsis* plants. Cold acclimated values of P700 oxidation of the Yukon and Shandong accessions were quite similar (Figure 9).

#### 2.6.2. Intersystem Electron Pool

**Figure 10:**
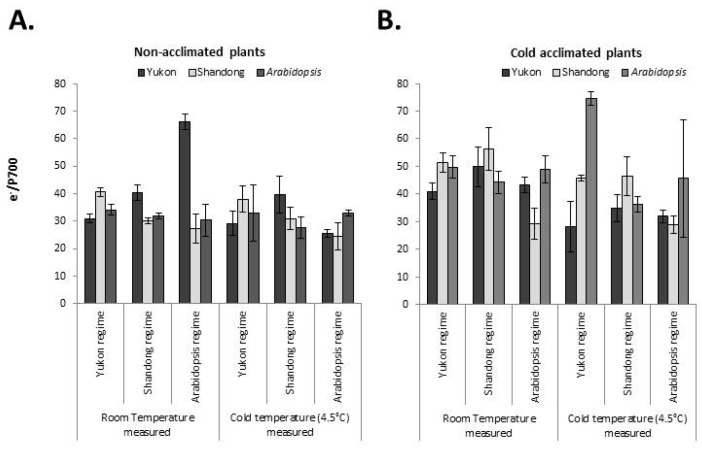
Pool size of electrons in the intersystem chain (e^−^/P700) for (**A**) non-acclimated and (**B**) cold acclimated *Eutrema* accessions and *Arabidopsis* developed under three different growth regimes. Yukon accession (■); Shandong accession (■); *Arabidopsis* (■). Plants were measured at room (20 °C) and cold (4.5 °C) temperatures. Values represent means ± SE (*n* = 4 to 9). SE = standard error.

The e^−^/P700 was found to be the product of complex interactions between the experimental accessions, growth regimes or measurement conditions. There were no consistent responses of the experimental accessions to measurement temperature, cold acclimation or growth regime for this parameter (Figure 10). Exceptionally higher values of e^−^/P700 were detected in NAWM Yukon plants in the *Arabidopsis* growth regime and CACM measured values of *Arabidopsis* plants in the Yukon growth regime. These observations were associated with apparent growth abnormalities. In the former case, Yukon plant growth was arrested due to a limitation of growth irradiance, while in the latter case, *Arabidopsis* plants displayed pale and stunted foliage as a combined effect of cold, longer photoperiod and high irradiance. A generalized scenario of combined analysis displayed significant effects of cold acclimation (*P* < 0.001) and measurement temperature (*P* = 0.041). However, these trends were complicated by the significant interaction of accessions with growth regime and measurement temperature. In general, cold acclimation resulted in an increase of the intersystem electron pool in Shandong (7% to 86% increase) and *Arabidopsis* (31% to 126% increases), while for Yukon plants this trend was not amenable for generalization due to interacting effect of growth regime and measurement temperature. Barring few exceptional observations, measurement at low temperature caused the lowering of e^−^/P700 in all three accessions across all growth regimes. The differences in the low temperature-measured values of the Yukon accession between the non-acclimated and cold acclimated plants were relatively smaller. The trend of the effect of growth irradiance on e^−^/P700 is variable between the experimental accessions. Yukon and *Arabidopsis* plants displayed interactions between growth regime, acclimation and measurement temperature, making it difficult to discern the effect of growth irradiance. However, for Shandong plants, e^−^/P700 values showed an increasing trend (10% to 90% increases) as the result of increased growth irradiance (Figure 10).

## 3. Discussion

### 3.1. Differential Photoprotective Stratagies Indicated by Pigmentation

Chlorophyll and carotenoids are integral components of the photosynthetic machinery. Chlorophyll content is the proxy indicator of photosynthetic competence of plants, while carotenoid content reflects photoprotective processes such as the xanthophyll cycle, resulting in the formation of NPQ. Therefore, leaf pigment composition can reflect the acclimation and adaptation strategies of plants in response to environmental factors that affect photosynthesis [48].

A combined analysis of various photosynthetic pigmentation parameters showed significant differences between the accessions across growth conditions shaped by individual environmental factors and their interplay. Significant two- or three-way interactions between the accessions, growth regimes and acclimation status indicated that Yukon, Shandong and *Arabidopsis* plants differentially respond to environmental variables. With a few exceptions arising from the interactions between the experimental factors, *Eutrema* accessions showed a significantly higher content of both chlorophyll and carotenoid pigments than *Arabidopsis* both on a per unit FW and unit LA basis across all growth regimes under both cold acclimated and non-acclimated conditions. All three accessions acclimated to low temperature by lowering the Chl:Car ratio, but *Eutrema* accessions reduced the chlorophyll content, while *Arabidopsis* increased the carotenoid content. Previous studies have shown that leaves of *Eutrema* contained approximately a 30% higher chlorophyll content than that of *Arabidopsis* and the pigment disparity between the accessions increased upon salinity treatment [45,46,49]. These results indicate that *Eutrema* accessions not only maintain greater photosynthetic and photoprotective potential than *Arabidopsis*, they also modulate photosynthetic and photoprotective strategies more dynamically in response to environmental conditions. These observations correspond well with the significantly higher capacity of *Eutrema* to oxidize P700 and have higher light saturation points with respect to Φ_PSII_, RETR_PSII_, 1-q_L_ and Φ_NPQ_ (see below). The increase in light saturation levels of PSII performance parameters due to cold acclimation (acclimation capacity) was also higher in *Eutrema*. These features reflect the extremophillic ecological background of *Eutrema* that requires a more dynamic mechanism to balance photosynthetic and photoprotective potentials, in contrast to the glycophytic adaptation of *Arabidopsis*.

Yukon and Shandong plants also showed differential pigment modulating properties in response to environmental variables. Shandong plants seemed to have the strategy of more abundant light interception and energy transformation, with a higher content of both chlorophyll and carotenoids per unit LA than that of Yukon plants. The higher pigment content of Shandong relates to the significantly higher RETR_PSII_ of this accession compared to that of Yukon accession. These accessions also differed in cold acclimation strategies in that Yukon had a significant increase Chl *a*:*b* due to cold acclimation across all growth regimes, but Shandong showed variable trends with no appreciable alteration in Chl *a*:*b*. This suggests that the Yukon accession tends to cold acclimate by reducing photosynthetic light harvesting, while the Shandong accession undergoes a proportionate decrease in both pigments (Chl *a*+*b*) during cold acclimation. These differences in cold acclimation strategies are also reflected in the differential trends of P700 oxidation in Yukon and Shandong plants (see below).

### 3.2. Similar Trends but Quantitative Differences in PSII Fluorescence Parameters

*Eutrema* and *Arabidopsis* plants grown under various conditions showed comparable trends in PSII performance indicators. As was anticipated, a generalized pattern of each accession across all growth conditions was that measurements at low temperature caused significant increases in excitation pressure with a concomitant down-regulation of Φ_PSII_ and RETR_PSII_, and consequently saturation of photosynthesis at lower irradiance levels. At the same time, the photosynthetic down-regulation was also associated with upregulation of NPQ parameters.

An examination of electron transport rate and pattern of energy partitioning revealed that photochemical down-regulation with concomitant upregulation of NPQ was a common manifestation of cold acclimation in both *Eutrema* accessions and *Arabidopsis*. At respective growth temperatures, the light response curves of cold acclimated plants positioned invariably at lower levels and light saturation occurred at lower PPFDs than that of non-acclimated control plants.

When plants were exposed to temperatures that contrasted with their respective growth temperatures, cold acclimated plants out-performed the non-acclimated counterparts with regard to PSII photochemistry. Exposure of cold acclimated plants to warm temperature (CAWM) resulted in the thermal augmentation of PSII performance characterized by the relaxation of excitation pressure, a greater fraction of excitation energy partitioned to photochemistry and a concomitant reduction of NPQ over a wide range of PPFDs. On the other hand, non-acclimated plants upon exposure to low measuring temperature (NACM) displayed a significant depression in the light response curves of photochemical indicators, suggesting the inhibition of photosynthesis. Growth regimes also triggered conspicuous effects on photosynthetic light response curves. Compared to the plants grown at low irradiance (*Arabidopsis* growth regime), the plants grown under higher irradiance (Yukon and Shandong growth regimes) underwent a down-shift in the trend of PSII excitation pressure coupled with upward shifts in light response curves for Φ_PSII_ and RETR_PSII_.

Amidst the common general trends of taxonomic responses to environmental variables, *Eutrema* and *Arabidopsis* plants differed from each other in the relative magnitude of PSII performance parameters. In most instances of the experimental settings, Shandong plants stood superior to the other plants, followed by the Yukon accession and then closely by *Arabidopsis*. This generalization was more applicable after the exclusion of results from the *Arabidopsis* growth regime that was proven to be a sub-optimal growth condition for the Yukon accession. Both the Yukon and Shandong accessions of *Eutrema* were able to cold acclimate without affecting their potential quantum efficiencies, while *Arabidopsis* plants acclimated to low growth temperature by lowering the F_v_/F_m_ by about 5% to 10%. These observations corroborate with an earlier finding that showed incomplete recovery of photosynthetic capacity of *Arabidopsis* upon cold acclimation [50]. In the range of experimental PPFDs, Shandong plants underwent a relatively larger fraction of excitation energy partitioning towards PSII photochemistry compared to that of Yukon and *Arabidopsis* plants, while the latter displayed more or less similar responses to each other. Compared to Yukon and *Arabidopsis* plants, the Shandong accession showed higher rates of RETR_PSII_ on the light response curve under all experimental conditions, with less intensity of RETR_PSII_ depression of non-acclimated plants due to cold shock (NACM) and a greater magnitude of thermal augmentation of RETR_PSII_ for cold acclimated plants due to exposure to warm measuring temperatures (CAWM). A recent comparative study also showed that Shandong had higher RETR_PSII_ than *Arabidopsis* under both normal and salt-stressed conditions [45]. Shandong plants also displayed less intensity of the effects of cold shock on PSII excitation pressure and NPQ parameters. On the other hand, the acclimative gain from the cold shock level (NACM) and thermal relaxation due to the warming effect (CAWM) were relatively lower in Shandong than that of *Arabidopsis*. These are the indication that Shandong has better photosynthetic stability. The Yukon and *Arabidopsis* exhibited contrasting effects of cold shock on NPQ parameters. The Yukon accession displayed highest sensitivity of overall NPQ while *Arabidopsis* showed highest sensitivity of q_O_ to the cold shock.

*Eutrema* and *Arabidopsis* plants also differed in photoinhibition of PSII. Yukon and Shandong accessions displayed relatively more stable values of F_v_/F_m_ in response to photoinhibitory treatments than *Arabidopsis* plants. When grown under higher irradiance non-acclimating conditions (250 μmol photons m^−2^ s^−1^), photoinhibitory responses of *Arabidopsis* were at par with *Eutrema*. However, when grown under lower irradiance non-acclimating conditions (100 μmol photons m^−2^ s^−1^), *Arabidopsis* showed greater susceptibility to photoinhibition than the *Eutrema* accessions. When grown under cold acclimating conditions, *Eutrema* accessions consistently displayed significantly less photoinhibition than *Arabidopsis*. Evidently *Eutrema* possess greater cold-acclimation capacity of photosynthetic machinery than that of *Arabidopsis*. The relative resistance to photoinhibition was found to be associated with carotenoid content in the leaves since these parameters were negatively correlated.

### 3.3. Differential Responses of Eutrema and Arabidopsis Plants to PSII Excitation Pressure

Photochemistry and the non-photochemical dissipation of energy are competing processes. A high degree of determination of regression of the photochemical and NPQ parameters on 1-q_L_ suggested the later to be an important link in photosynthetic processes. The scatter plots and regression parameters of Shandong, Yukon and *Arabidopsis* plants showed that Shandong is quantitatively more responsive to excitation pressure for Φ_PSII_ and Φ_NPQ_, while in *Arabidopsis* q_O_ shows more involvement. It has already been postulated that the energy partitioning through these competing pathways is regulated by the environmental signals perceived by photosynthesis itself through the redox state of photosynthetic electron transport components [3,51].

The above observations led to the consideration that excitation pressure may serve as the unifying determinant of the energy partitioning. To examine the pattern of energy partitioning in response to excitation pressure, the results of energy fractionation from different experimental conditions were plotted against the excitation pressure. The portrait of energy partitioning against excitation pressure revealed a complex nature of energy fractionation that cannot be ascribed to short-term changes in excitation pressure. However, the results suggested that an excitation pressure of around 0.50 is critical for relative predominance of Φ_PSII_ or Φ_NPQ_. When measured at growth irradiance, the intersection of Φ_PSII_ or Φ_NPQ_ curves occurred between excitation pressures of 0.49 and 0.62 in non-acclimated plants and between 0.42 and 0.56 in cold acclimated plants. *Arabidopsis* plants displayed relatively lower Φ_PSII_ or higher Φ_NPQ_ at a given excitation pressure than *Eutrema* accessions. At reciprocal measurement temperatures, cold acclimated plants displayed a higher sensitivity of energy partitioning patterns to the changes in excitation pressure than the non-acclimated plants. This suggests that cold acclimation results in the development of more responsive mechanisms to detect changes in environmental conditions, thereby enabling them to adjust the pattern of excitation energy partitioning. Considering the extremophillic adaptation of *Eutrema* in contrast with the glycophytic adaptation of *Arabidopsis*, it was anticipated that *Eutrema* would possess better resiliency of PSII performance especially under low temperature conditions. However, Shandong plants only displayed minor quantitative differences from *Arabidopsis*, while Yukon appeared fairly similar to *Arabidopsis*. This may be due to the fact that the treatments imposed in the experiments were within the adaptive range of *Arabidopsis*.

### 3.4. Yukon, Shandong and Arabidopsis Plants Show Divergent Trends in PSI Performance

PSI has crucial role in balancing the phosphorylating and reducing potentials of cellular metabolism [52]. Oxidation of P700, an indicator of PSI activity was measured as the far-red light induced absorbance change at 820 nm (Δ*A*_820_). The redox state of P700 reflects the metabolic condition of chloroplast including the availability of the electron acceptors such as NADP^+^, the extent of alternative electron transfer pathways around PSI, the redox state of the ferredoxin pool and electron transfer from PSII [53]. These properties, in turn, are a function of genotype, environmental variables and their interactions. Oxidized P700 is reduced by electrons mainly originating from the photooxidation of water which are conveyed through linear electron transport via PSII, the plastoquinone pool and plastocyanin. The intersystem electron pool is also fed by other pathways [54]. These properties constitute components of photosynthetic adjustment strategies of plants in response to environmental conditions [55].

Yukon, Shandong and *Arabidopsis* plants displayed divergent patterns of P700 oxidation with interactions between the accessions and environmental variables. The difference in the P700 oxidation was accompanied with active electron transport from PSII, as evidenced by a full reduction of oxidized P700 from MT flashes. Except for the case of Yukon plants in the light-limited condition of the *Arabidopsis* growth regime, *Eutrema* accessions displayed a significantly higher amount of oxidizable P700 than *Arabidopsis* under all conditions and acclimation status. Under non-acclimated conditions, both accessions of *Eutrema* and *Arabidopsis* showed a growth irradiance-dependent response of P700 oxidation. However, both accessions of *Eutrema* contrasted with *Arabidopsis* in having a higher magnitude of growth irradiance response. With the increase in irradiance from 100 μmol photons m^−2^ s^−1^ (*Arabidopsis* growth regime) to 250 μmol photons m^−2^ s^−1^ (Yukon and Shandong growth regimes), the *Eutrema* accessions responded with a minimum of an 82% increase in P700 oxidation whereas the corresponding increase in P700 oxidation of *Arabidopsis* was approximately 28%. Non-acclimated Shandong plants consistently displayed higher amounts of oxidized P700 than both Yukon and *Arabidopsis* plants. The Yukon accession significantly outperformed *Arabidopsis* under a growth irradiance of 250 μmol photons m^−2^ s^−1^. However, it is notable that Yukon had the lowest oxidizable P700 in the *Arabidopsis* growth regime that presumably barely met the light compensation point of this accession, resulting in stagnant growth and eventual collapse of plants after 4 weeks of germination (data not shown). This shows an interesting relationship between P700 oxidation and plant growth. In fact, an earlier study has shown the relationship between the oxidizable P700 and CO_2_ assimilation in plants [53]. Yukon and Shandong plants also contrasted each other and with *Arabidopsis* plants in the cold acclimated response of P700. Although cold acclimated *Arabidopsis* had lower oxidizable P700 than *Eutrema*, it displayed the greatest acclimatory change in this parameter due to cold acclimation. The Yukon accession also underwent a significant increase in P700 oxidation as a cold acclimatory response. However, Shandong showed a growth irradiance-dependent acclimation pattern. In this accession, there was marked increase in oxidizable P700 as a result of acclimation in the *Arabidopsis* growth regime (low irradiance), but no acclimatory response in the Yukon and Shandong growth regimes (higher irradiance). This is presumably due to enhancement of plastid terminal oxidase (PTOX) activity of Shandong at higher irradiance, rendering acclimatory adjustment in PSI unnecessary in the given environmental conditions. This argument can be supported by a previous study that demonstrated salinity treatment triggered a significant up-regulation of PTOX with a concomitant increase in RETR_PSII_ in Shandong, while in *Arabidopsis* there was no such up-regulation of PTOX and RETR_PSII_ was actually reduced [45]. In the current study, higher irradiance could have triggered such a response in the Shandong accession. Presumably, the irradiance was not high enough for triggering PTOX or other alternative mechanisms in Yukon, as this accession had more sensitive growth plasticity to irradiance suggesting a higher irradiance requirement for optimal growth than to that of Shandong and *Arabidopsis* plants.

For the pool size of electrons in the intersystem chain (e^−^/P700), there were no consistent responses of the experimental accessions to measurement temperature, cold acclimation or growth regime. In general, Shandong plants showed a trend of increasing e^−^/P700 with increase in irradiance and also due to cold acclimation. In the Shandong accession, there was some correspondence between e^−^/P700 and RETR_PSII_ that explains partly what results in the increased e^−^/P700 size. However, Yukon plants did not display any definite trend of e^−^/P700 in response to environmental variables, while *Arabidopsis* responded to cold acclimation with an increase in e^−^/P700, without any definite trend across the growth regimes. Moreover, Yukon and *Arabidopsis* plants that had perturbed phenotypes observed in two contrasting growth regimes and acclimation status showed an exceptional escalation of e^−^/P700 for contrasting measurement temperatures. In Yukon plants, those observations were associated with warm temperature measurements of the plants from the non-acclimated *Arabidopsis* growth regime where plant growth was severely arrested due presumably to a deficit of irradiance to meet the metabolic demand of the plants. In *Arabidopsis*, on the other hand, the exceptional results were associated with low temperature measurement of cold acclimated plants from the Yukon growth regime, where plant phenotypes were chlorotic in nature. In fact, cold acclimated *Arabidopsis* had significantly lower chlorophyll contents in the Yukon growth regime compared to other growth regimes. These results showed that there was a complex interaction between the accessions and environmental variables that determine the relative predominance of electron flux in the intersystem pool of electrons.

## 4. Materials and Methods

### 4.1 Plant Material and Growth Conditions

Seeds of *Arabidopsis thaliana* (L.) Heynh. (accession Columbia, Col-0, stock no. CS60000) and *Eutrema salsugineum* (Pall.) Al-Shehbaz Shehbaz & Warwick (Shandong accession, stock no. CS22504 and Yukon accession, stock no. CS22664) were obtained from the Arabidopsis Biological Resource Center (ABRC, The Ohio State University, Columbus, OH, USA) were germinated and maintained in controlled environment growth chambers (Model PGR15; Conviron, Winnipeg, MB, Canada) using the non-acclimating growth conditions described in Table 1 [39]. These are referred to as Yukon, Shandong and *Arabidopsis* growth regimes.

The calculation of daily photon receipt (DPR) was performed as follows: the instantaneous units of irradiance as μmol photons m^−2^ s^−1^ were converted to mol photons m^−2^ d^−1^, where d denotes day.

DPR = (μmol photons m^−2^ s^−1^)/1,000,000 × photoperiod × 60 × 60

The cumulative daily average temperature (DAT) was estimated as:

DAT = [(Light time temperature × light period)/24] + [(dark time temperature × dark period)/24]

For cold acclimation, separate flats of plants maintained under these conditions were shifted to a 5/4 °C (day/night) temperature with the same irradiance and photoperiod as the non-acclimated plants in all growth regimes (Table 1). Photosynthetic parameters of non-acclimated *Eutrema* and *Arabidopsis* were measured after 4 weeks and 3 weeks of sowing respectively and those of cold acclimated plants were measured after 3-weeks of cold acclimation.

It is noteworthy that the poor growth of the Yukon accession under the non-acclimating, *Arabidopsis* growth regime resulted in a lack of suitably sized leaves for certain chlorophyll fluorescence measurements. This was circumvented in some cases by using fluorescence imaging or the construction of leaf discs comprised of multiple leaves. However, upon cold acclimation for 3 weeks, the Yukon leaves attained measurable growth allowing for subsequent analyses.

### 4.2. Chlorophyll a Fluorescence

#### 4.2.1. Steady-State Fluorescence Quenching

Chlorophyll steady-state fluorescence quenching characteristics were determined in vivo using detached leaves under saturated CO_2_ conditions using a XE-PAM xenon-pulse amplitude modulation fluorometer (Heinz Walz GmbH, Effeltrich, Germany) as described in detail previously [56]. Measurements were made using a Hansatech leaf-disc chamber (LD2/3; Hansatech Instruments Ltd., King’s Lynn, Norfolk, UK) modified with an adapter (LD/FA; Hansatech) to accept the PAM fibreoptic. The fluorescence nomenclature and derivation of parameters were adopted from [57,58,59].

The temperature inside the chamber was maintained by a refrigerated circulating water bath (model RC6 CS; Lauda Dr. R. Wobser GmbH and Co., KG, Lauda-Königshofen, Germany) and matched that of the growth temperature for non-acclimated and cold acclimated conditions in each growth regime. Measurements were made at reciprocal temperatures so that there were four measuring conditions in each treatment; they are: non-acclimated plants measured at the growth temperature (non-acclimated warm-measured, NAWM; control), non-acclimated plants measured at low temperature (non-acclimated cold-measured, NACM; cold shock), cold acclimated plants measured at the growth temperature (cold acclimated cold-measured, CACM; cold acclimative), and cold acclimated plants measured at non-acclimating temperature (cold acclimated warm-measured, CAWM; thermal relaxation/augmentation). This design allowed for the dissection of the effects of cold shock, cold acclimation and thermal relaxation/augmentation of photosynthetic parameters by the comparison of measurements as follows:

Cold shock effect with respect to corresponding control = (NAWM − NACM)/NAWM

Cold acclimative effect with respect to corresponding cold shock = (CACM − NACM)/NACM

Thermal relaxation/augmentation effect = (CAWM − CACM)/CACM

These calculations were performed over the range of actinic irradiance where the tissue had equilibrated to the cuvette temperature to experience the cold shock or warming effect. These were 133 to 1790 μmol photons m^−2^ s^−1^ for 1-q_L_ and RETR_PSII_, and 670 to 1790 µmol photons m^−2^ s^−1^ for q_O_.

Leaves were dark-adapted for 15 min prior to the onset of measurement which was determined empirically. Further increases in dark adaptation time did not result in any changes in fluorescence parameters. Minimal fluorescence in the dark-adapted state (F_o_) was determined by subjecting the leaf sample to ms probe flashes from a xenon-arc measuring beam. A saturating pulse (6500 μmol photons m^−2^ s^−1^) of light for 800 ms was used to determine the maximal fluorescence (F_m_) in the dark-adapted state. The leaves were then exposed to a series of actinic PPFDs of various intensities from 55 to 1790 μmol photons m^−2^ s^−1^ until a stable, steady-state level of fluorescence (F′) was achieved, approximately 10 min after switching to the next higher light level. Application of another saturating pulse gave the maximal fluorescence (F_m_′) in light-adapted state. Minimal fluorescence in the light-adapted state (F_o_′) was determined immediately after turning off the actinic source in the presence of far-red (FR) background light for 4 s to ensure maximal oxidation of PSII electron acceptors. WinControl software (ver 1.93; Heinz Walz) was used in conjunction with the PAM-data acquisition system (PDA-100; Heinz Walz) to control the timing, settings and trigger signals for the various actinic and saturating pulse light sources. Fluorescence traces were captured and analysed using the WinControl software.

The maximum quantum efficiency of PSII photochemistry (F_v_/F_m_ = (F_m_ − F_o_)/F_m_) and the coefficient of basal fluorescence quenching (q_O_ = (F_o_ − F_o_′)/F_o_) were calculated according to [60] and [61] respectively. PSII excitation pressure was expressed as 1-q_L_ and calculated as 1 − (F_q_′/F_v_′) × (F_o_′/F′), where F_q_′ = F_m_′ − F′ [58]. The relative non-cyclic electron transport rate through PSII (RETR_PSII_) was determined as Φ_PSII_ × I × 0.42, where I is the incident PPFD on the leaf and 0.42 is the product of the spectral absorbance of the leaf (84%) and the fraction of incident photons that are absorbed by PSII (50%) [62].

The partitioning of absorbed light energy into PSII photochemistry and non-photochemical processes was estimated according to the model proposed by [57]. In this model, the fraction of energy utilized to drive PSII photochemistry (PSII operating efficiency) is estimated as Φ_PSII_ ((F_m_ − F′)/F_m_′ = F_q_′/F_m_′), the fraction of energy dissipated as light-dependent non-photochemical quenching is estimated as Φ_NPQ_ (((F_m_ − F_m_′)/F_m_) × (F′/F_m_′) = (F′/F_m_′) − (F′/F_m_)) and the efficiency of constitutive non-photochemical energy dissipation and fluorescence is estimated as Φ_NO_ (F′/F_m_), hence Φ_PSII_ + Φ_NPQ_ + Φ_NO_ = 1.

#### 4.2.2. Chlorophyll Fluorescence Imaging

Chlorophyll fluorescence imaging was used to monitor photoinhibition and recovery in whole plants (see below) using a commercially available modulated imaging fluorometer (FluorCam; Photon System Instruments, Brno, Czech Republic) as described in detail by [56] except that only numerical data was acquired. Plants were dark-adapted for 15 min prior to measurement and the F_v_/F_m_ ratio with values integrated for the entire plant.

### 4.3. Photoinhibitory Treatments and Recovery

For photoinhibitory treatments, whole plants were exposed to 1750 μmol photons m^−2^ s^−1^ in a cold room set at 2 °C for 4 h using high pressure sodium bulbs (Sylvania Lumalux LU400/Eco, Osram Sylvania Products Inc., Manchester, NH, USA). Temperature at the leaf surface never exceeded 7 °C. The plants were allowed to recover at low light (30 μmol photons m^−2^ s^−1^) at 22 °C for 24 h. Photoinhibition and recovery were quantified by assessment of changes in F_v_/F_m_ as described above.

### 4.4. Photosystem I Spectroscopy

The relative redox state of P700 was estimated in vivo as the light induced change in absorbance at 820 nm (Δ*A*_820_) under ambient CO_2_ conditions using a PAM-101 modulated fluorometer (Heinz Walz) as described in detail previously by [63]. Plants for each treatment were measured at room (20 °C) and cold (4.5 °C) temperatures to represent the growth temperatures for non-acclimated and cold acclimated conditions in each growth regime. Measurements were also done at reciprocal temperatures. The temperature was maintained by a refrigerated circulating water bath (Lauda Dr. R. Wobser).

Briefly, FR light (λ_max_ 735 nm) at an intensity of approximately 70 μmol photons m^−2^ s^−1^ was applied on the adaxial side of the leaf in a custom designed cuvette modified to accept the PAM fibre optic. The redox state of P700 was evaluated as Δ*A*_820_ due to the formation of the cation radical (P700^+^). After the P700^+^ signal reached a steady-state level, single turnover (ST) saturating flashes (half peak width 14 μs) and multiple turnover (MT) saturating flashes (50 ms) were applied for the transient reduction of P700^+^ in the presence of the background FR irradiance. WinControl software (version 1.93; Heinz Walz) was used in conjunction with the PAM-data acquisition system (PDA-100; Heinz Walz) to control the timing, settings and trigger signals for the various light sources. Traces were captured using the WinControl software and data files were exported to MicoCal Origin (version 6.0; MicroCal Software Inc., Northampton, MA, USA) for plotting, smoothing and integration of the areas under the curves representing the reduction of oxidized P700 due to the ST and MT turnover flashes. The apparent size of the intersystem electron donor pool to PSI (e^−^/P700) was estimated in vivo as a ratio of the area associated with the MT and ST flashes (MT area/ST area) as described by [54].

### 4.5. Photosynthetic Pigment Determination

Plant pigments were extracted from leaves in 80% (*v*/*v*) acetone by grinding in a mortar and pestle followed by centrifugation for 5 minutes at 4500 rpm. Pigments were quantified spectrophotometrically (SmartSpec Plus; Bio-Rad Laboratories, Hercules, CA, USA) according to the equations of [64] and expressed on a leaf fresh weight (FW) or leaf area (LA) basis. Measurements of leaf FW and leaf area were carried out using an analytical balance (Mettler Toledo, Columbus, OH, USA) and leaf area meter (LI-COR Inc., Lincoln, NE, USA).

### 4.6. Experimental Design and Statistical Analyses

The experiments were conducted in completely randomized design and measurements were replicated three or more times. A planting tray containing 36 cells constituted a block in which three experimental accessions were randomly assigned to the group of 12 cells. One tray was considered one replicate. Data were analysed by using descriptive statistics, correlation and analysis of variance (ANOVA) techniques with the aid of Microsoft Excel 2007 (Microsoft Corporation, Redmond, Washington, DC, USA), SigmaPlot 12 (Systat Software, Inc., San Jose, CA, USA), and Minitab version 15 (Minitab Inc., State College, PA, USA). Means of the experimental factors were grouped by Fisher’s individual error rate at significance level of 0.05.

## 5. Conclusions

Photosynthesis is highly sensitive to environmental variability and a prime target of abiotic stress. Adaptation to prevailing growth conditions requires dynamic and flexible modulation of the photosynthetic machinery in response to environmental cues, including a tightly regulated energy balancing mechanism of photosynthetic metabolism. In this study, we have performed comparative experiments aimed to characterize basic photosynthetic properties of the Yukon and Shandong accessions of *Eutrema* as well as *Arabidopsis* grown under different growth regimes. Because of their extremophillic nature, it was expected that *Eutrema* would possess a differential capacity to modulate photosynthetic responses to PSII excitation pressure (light and low temperature) in comparison to *Arabidopsis*.

### 5.1. Comparisons between Eutrema and Arabidopsis

Our analyses revealed that photosynthetic parameters differed intraspecifically between the two accessions of *Eutrema* and *Arabidopsis*. *Eutrema* accessions showed a significantly higher content of both chlorophyll and carotenoid pigments across all growth regimes under both acclimated and non-acclimated conditions in comparision to *Arabidopsis*. Significant interactions between accessions, growth regime and acclimation status for photosynthetic pigments content and ratios suggests differential photosynthetic acclimation responses of the accessions to growth temperature, irradiance and photoperiod. *Eutrema* accessions were able to cold acclimate without an apparent effect on F_v_/F_m_, while *Arabidopsis* cold acclimates with a down-regulation of F_v_/F_m_. The processes modulating F_v_/F_m_, such as growth conditions, are prominent strategies of cold acclimation in both *Eutrema* and *Arabidopsis*. These accessions showed differential physiological plasticity to growth temperature and irradiance. However, growth irradiance seemed to be an important determinant of the tolerance of plants to photoinhibition of photosynthesis. In general, plants grown with higher irradiance experienced less photoinhibition than those grown under lower irradiance. *Eutrema* accessions possessed greater constitutive tolerance to photoinhibition and were better able to attained energy balancing during cold acclimation than *Arabidopsis* plants. Photoinhibition at low temperature was reversible in all accessions, suggesting no differences in the effectiveness of repair of the photosynthetic apparatus. All accessions demonstrated a full recovery from photoinhibition after the removal of the stress.

Fluorescence quenching analyses demonstrated a similar trend in responses, although quantitative differences were evident. Plants grown under a higher DPR presented lower values of 1-q_L_ when exposed to momentary high irradiance and/or cold temperature. In the range of low to saturating irradiance levels, *Eutrema* plants showed more stable 1-q_L_ than *Arabidopsis* when exposed to cold shock and/or thermally relaxing conditions. Both *Eutrema* and *Arabidopsis* plants acclimated under low temperatures by down-regulating photosynthesis. However, *Eutrema* showed greater stability in RETR_PSII_ than *Arabidopsis* in the range of low to saturating irradiance levels upon the exposure to cold shock and/or thermally relaxing conditions. In all accessions, cold acclimation elicited a greater dissipation of excitation energy from the light-harvesting antenna of PSII (q_O_). Such dissipation of excitation energy was higher in non-acclimated *Eutrema* than in non-acclimated *Arabidopsis*, and contrastingly lower in cold acclimated *Eutrema* than in cold acclimated *Arabidopsis*. Growth conditions exerting higher excitation pressure (high irradiance and low temperature) resulted in the energy partitioning in favour of NPQ at the expense of PSII photochemistry. However, growth conditions with higher irradiance proved more favorable for the development of more resilient photochemistry and generally, energy partitioning in *Eutrema* was more favourable for photochemistry than that of *Arabidopsis* plants. Moreover, cold acclimation of photosynthesis resulted in only partial recovery of photochemistry in both *Arabidopsis* and *Eutrema* plants.

A property that both *Eutrema* accessions shared while contrasting with *Arabidopsis* was that of significantly higher PSI activity in all growth regimes. Both accessions of *Eutrema* outperformed *Arabidopsis* plants for P700 oxidation in all environmental conditions that meet the minimal growth requirement of the plants. Cold acclimation under higher DPR conditions (higher excitation pressure) significantly enhanced the P700 oxidizing capacity in *Eutrema*, but *Arabidopsis* underwent a depression in P700 oxidizing capacity due to cold acclimation. Thus, it appears mitigation of PSI limitation may play a prominent role in the mechanism of photosynthetic acclimation to high light and low temperature, ensuring PSI is not acceptor-limited and conferring protection to PSII by oxidation of the plastoquinone pool. While not examined in this study, the role of alternative electron sinks may also contribute to the acclimatory adjustments enabling adaptation to harsh environmental conditions as have been shown previously for both *Eutrema* and *Arabidopsis* [21,45].

### 5.2. Comparisons between Eutrema Accessions

Numerous lines of evidence substantiate that geographical separation of a population leads to the development of photosynthetic strategies as dictated by the local environmental conditions [36,37]. Having evolved in contrasting ecophysiological backgrounds, the Yukon and Shandong accessions of *Eutrema* as well as *Arabidopsis* presumably respond differentially to environmental variability.

Surprisingly, it was the Shandong accession of *Eutrema* which exhibited better photosynthetic plasticity and not the Yukon accession as we would have predicted based on its natural habitat which is much harsher than that of Shandong [34]. Both *Eutrema* accessions acclimated under low temperatures with the down-regulation of photosynthesis. In the range of low to saturating irradiance, Shandong plants showed a greater stability in 1-q_L_ and RETR_PSII_ than Yukon plants when exposed to cold shock and thermally relaxing conditions. Energy partitioning in the Shandong accession of *Eutrema* was more favourable for photochemistry than that of Yukon accession. While P700 oxidation was greater in *Eutrema* overall, non-acclimated Shandong plants consistently displayed higher amounts of oxidized P700 in comparison to Yukon plants. Cold acclimation under higher DPR conditions (higher 1-q_L_) significantly enhanced the P700 oxidizing capacity in Yukon followed distantly by Shandong.

While both accessions respond in a manner that allows more efficient photosynthetic acclimation than *Arabidopsis*, the mechanisms utilized are quite different and likely a result of adaptation to the natural conditions of these accessions. For the Yukon accession, we propose that as a result of growth in an environment where a plant must deal with multiple stress simultaneously (high 1-q_L_) a mechanism of rapid response is required. This is activated only in response to stress conditions and along with already higher levels of constitutive tolerance facilitate survival. In the Yukon accession this results in excitation partitioning to more protective processes which likely involve photochemical and non-photochemical strategies. In contrast, the Shandong accession is much more stable in its photosynthetic response to stress and responds more slowly, partitioning excess excitation to photochemistry. Thus, it is possible that at least for photosynthetic acclimation, the Yukon accession has ‘locked in’ mechanisms of adjustment that permit rapid responses to environmental change that ensure survival, albeit at the expense of the enhanced flexibility exhibited by the Shandong accession.

## Figures and Tables

**Table 1:** Growth regimes used in this study.

Growth Regime	Acclimation Status									
	Non-Acclimated					Cold Acclimated				
	Temperature (Light/Dark)	Photoperiod (Light/Dark)	PPFD (μmol photons m^−2^ s^−1^)	DPR (μmol photons m^−2^)	DAT (°C)	Temperature (Light/Dark)	Photoperiod (Light/Dark)	PPFD (μmol photons m^−2^ s^−1^)	DPR (μmol photons m^−2^)	DAT (°C)
Yukon	22/10 °C	21/3 h	250	18.90	20.5	5/4 °C	21/3 h	250	18.90	4.9
Shandong	22/19 °C	16/8 h	250	14.40	21.0	5/4 °C	16/8 h	250	14.40	4.7
*Arabidopsis*	20/20 °C	16/8 h	100	5.75	20.0	5/4 °C	16/8 h	100	5.75	4.7

See Section 4.1. Plant Material and Growth Conditions for calculations of DPR and DAT. DAT = daily average temperature; DPR = daily photon receipt; PPFD = photosynthetic photon flux density.

**Table 2:** Comparison of photosynthetic leaf pigmentation in the Yukon and Shandong accessions of *Eutrema* and *Arabidopsis* at three different growth regimes under non-acclimating and cold acclimating conditions. Values followed by small case letters along columns are significant within growth regime and the capital letters along the rows denote the significant difference of an accession across the acclimation state.

Parameter	Taxa (Accession)	Growth Regime					
		Yukon		Shandong		*Arabidopsis*	
		Non-Acclimated	Cold Acclimated	Non-Acclimated	Cold Acclimated	Non-Acclimated	Cold Acclimated
**ChlFW (μg mg^−1^)**	*Eutrema* (Yukon)	2.4 ± 0.07 ^a,A^	1.9 ± 0.08 ^a,B^	2.4 ± 0.10 ^a,A^	1.9 ± 0.05 ^a,B^	2.4±0.29 ^a,A^	2.2 ± 0.04 ^a,A^
	*Eutrema* (Shandong)	2.2 ± 0.02 ^a,A^	1.6 ± 0.03 ^b,B^	2.2 ± 0.11 ^a,A^	1.6 ± 0.02 ^b,B^	2.5±0.03 ^a,A^	1.1 ± 0.21 ^c,B^
	*Arabidopsis* (Columbia)	1.3 ± 0.05 ^b,A^	1.3 ± 0.06 ^c,A^	1.2 ± 0.07 ^b,A^	1.5 ± 0.03 ^c,A^	1.7±0.05 ^b,A^	1.6 ± 0.07 ^b,A^
**CarFW (μg mg^−1^)**	*Eutrema* (Yukon)	0.36 ± 0.009 ^a,B^	0.46 ± 0.010 ^a,A^	0.40 ± 0.005 ^a,B^	0.49 ± 0.012 ^a,A^	0.41 ± 0.047 ^a,A^	0.48 ± 0.022 ^a,A^
	*Eutrema* (Shandong)	0.43 ± 0.003 ^a,A^	0.35 ± 0.010 ^b,B^	0.34 ± 0.016 ^b,A^	0.37 ± 0.008 ^b,A^	0.38 ± 0.008 ^a,A^	0.30 ± 0.053 ^b,A^
	*Arabidopsis* (Columbia)	0.23 ± 0.010 ^b,B^	0.33 ± 0.010 ^b,A^	0.20 ± 0.013 ^c,B^	0.29 ± 0.012 ^c,A^	0.29 ± 0.021 ^b,A^	0.33 ± 0.013 ^b,A^
**ChlLA (μg cm^−2^)**	*Eutrema* (Yukon)	64.5 ± 5.22 ^a,A^	63.3 ± 1.35 ^b,A^	64.1 ± 2.45 ^a,A^	65.7 ± 4.08 ^a,A^	39.0 ± 2.2 ^b,B^	62.0 ± 2.48 ^a,A^
	*Eutrema* (Shandong)	72.2 ± 2.69 ^a,A^	71.3 ± 2.67 ^a,A^	71.3 ± 4.05 ^a,A^	78.1 ± 4.82 ^a,A^	61.7 ± 1.84 ^a,A^	32.8 ± 5.80 ^b,B^
	*Arabidopsis* (Columbia)	36.1 ± 2.38 ^b,A^	38.3 ± 2.35 ^c,A^	26.8 ± 1.26 ^b,B^	46.8 ± 1.46 ^b,A^	32.9 ± 1.26 ^c,B^	42.6 ± 1.30 ^b,A^
**CarLA (μg cm^−2^)**	*Eutrema* (Yukon)	9.9 ± 0.64 ^b,B^	15.4 ± 0.58 ^a,A^	10.8 ± 0.64 ^a,B^	17.3 ± 0.57 ^a,A^	6.7 ± 0.32 ^b,B^	13.5 ± 0.36 ^a,A^
	*Eutrema* (Shandong)	14.1 ± 0.44 ^a,A^	15.4 ± 0.43 ^a,A^	11.2 ± 0.73 ^a,B^	18.1 ± 1.16 ^a,A^	9.4 ± 0.14 ^a,A^	8.9 ± 1.47 ^b,A^
	*Arabidopsis* (Columbia)	6.5 ± 0.34 ^c,B^	9.5 ± 0.43 ^b,A^	4.4 ± 0.24 ^b,B^	9.0 ± 0.27 ^b,A^	5.5 ± 0.19 ^c,B^	8.7 ± 0.23 ^b,A^
**Chl *a*:*b***	*Eutrema* (Yukon)	3.1 ± 0.13 ^c,B^	4.9 ± 0.50 ^a,A^	3.3 ± 0.29 ^a,B^	4.8 ± 0.06 ^a,A^	2.8 ± 0.15 ^b,A^	3.8 ± 0.39 ^a,A^
	*Eutrema* (Shandong)	4.7 ± 0.12 ^a,A^	4.2 ± 0.10a ^b,A^	3.6 ± 0.20 ^a,B^	4.8 ± 0.22 ^a,A^	3.2 ± 0.14 ^b,A^	2.2 ± 0.30 ^b,B^
	*Arabidopsis* (Columbia)	3.9 ± 0.05 ^b,A^	3.7 ± 0.37 ^b,A^	4.0 ± 0.08 ^a,A^	3.3 ± 0.24 ^b,B^	3.9 ± 0.22 ^a,A^	4.1 ± 0.11 ^a,A^
**Chl:Car**	*Eutrema* (Yukon)	6.5 ± 0.21 ^a,A^	4.1 ± 0.16 ^a,B^	6.0 ± 0.28 ^a,A^	3.8 ± 0.14 ^b,B^	5.9 ± 0.41 ^a,A^	4.6 ± 0.29 ^a,A^
	*Eutrema* (Shandong)	5.1 ± 0.04 ^b,A^	4.6 ± 0.12 ^a,A^	6.4 ± 0.27 ^a,A^	4.3 ± 0.10 ^b,B^	6.6 ± 0.16 ^a,A^	3.7 ± 0.20 ^b,B^
	*Arabidopsis* (Columbia)	5.5 ± 0.23 ^b,A^	4.0 ± 0.24 ^a,B^	6.1 ± 0.05 ^a,A^	5.2 ± 0.28 ^a,A^	6.0 ± 0.28 ^a,A^	4.9 ± 0.08 ^a,B^

Values represent means ± SE (*n* = 3 to 5). The data were analysed using ANOVA and the means were separated using Fisher’s individual error rate at significance level of 0.05. Data are expressed on a leaf FW (FW) or a leaf area basis (LA). ANOVA = analysis of variance; Chl = chlorophyll; Car = carotenoid; SE = standard error.

**Table 3:** Effect of cold shock, cold acclimation and thermal relaxation/augmentation as the fraction of corresponding values of photosynthetic parameters for *Eutrema* accessions and *Arabidopsis.*

Parameters	Cold Shock Effect (NAWM-NACM)/NAWM			Cold Acclimative Effect (CACM-NACM)/NACM			Relaxation/Augmentation Effect (CAWM-CACM)/CACM		
	Yukon	Shandong	*Arabidopsis*	Yukon	Shandong	*Arabidopsis*	Yukon	Shandong	*Arabidopsis*
1-q_L_	−0.51	−0.42	−0.51	−0.17	−0.13	−0.20	−0.58	−0.48	−0.52
RETR_PSII_	0.54	0.49	0.57	0.34	0.32	0.40	1.05	1.18	0.94
q_O_	−0.39	−0.26	−0.99	0.16	0.13	0.30	−0.50	−0.52	−0.41

Values are averages from the Yukon and Shandong growth regimes. Results from the *Arabidopsis* growth regime were excluded due to incomplete data sets for the Yukon accession. Negative signs before the values indicate the direction of the treatment effect on the specific parameters. Magnitudes can be interpreted in the absolute terms. CACM = cold acclimated cold measured; CAWM = cold acclimated warm-measured; RETR_PSII_ = relative non-cyclic electron transport rate through PSII; NACM = non-acclimated cold-measured; NAWM = non-acclimated warm-measured; PSII = photosystem II; q_O_ = basal fluorescence quenching coefficient; 1-q_L_ = PSII excitation pressure.

## Supplementary Materials

Supplementary Materials can be found at www.mdpi.com/2223-7747/6/3/32/s1.
