# Supplementary figures and images for: Differential Mechanisms of Photosynthetic Acclimation to Light and Low Temperature in Arabidopsis and the Extremophile Eutrema salsugineum
Source: Plants (Basel). 2017 Aug 9;6(3):32. doi: 10.3390/plants6030032 (PMC5620588; doi:10.3390/plants6030032)

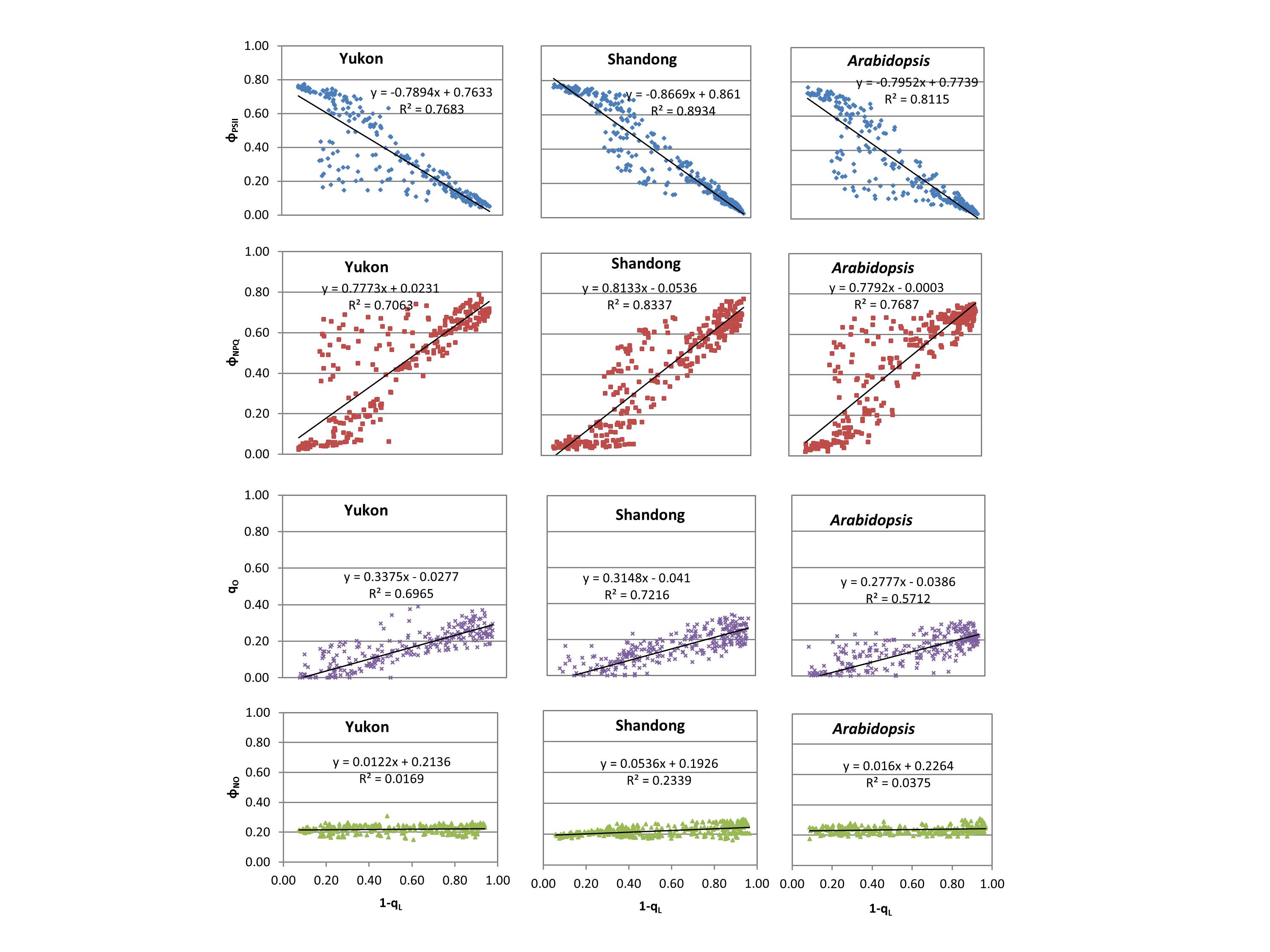

Supplement: Supplementary file 1 [file plants-06-00032-s001.zip › plants-206561-Supplementary Materials/Fig S1.jpg]

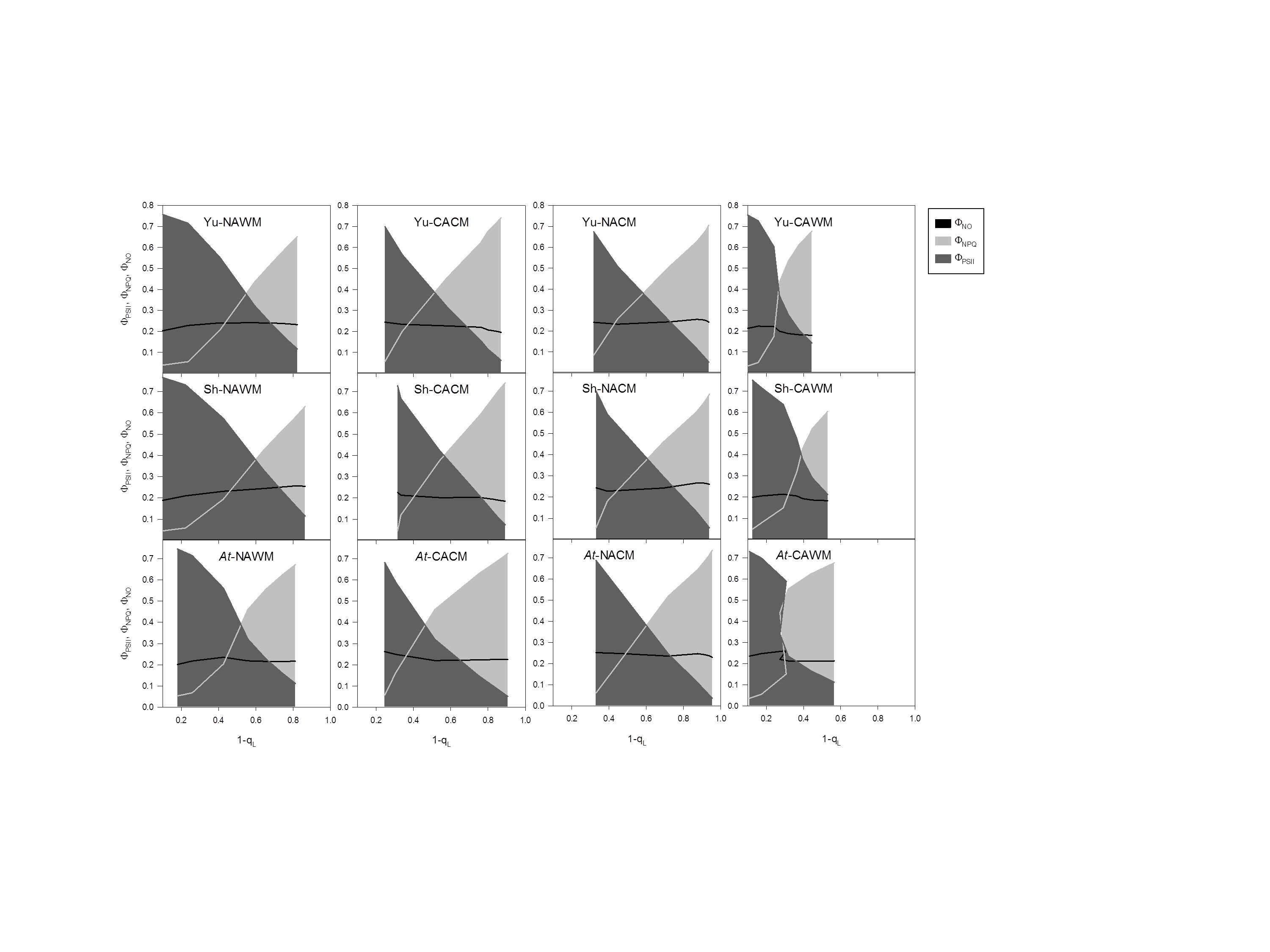

Supplement: Supplementary file 1 [file plants-06-00032-s001.zip › plants-206561-Supplementary Materials/Fig S2.JPG]

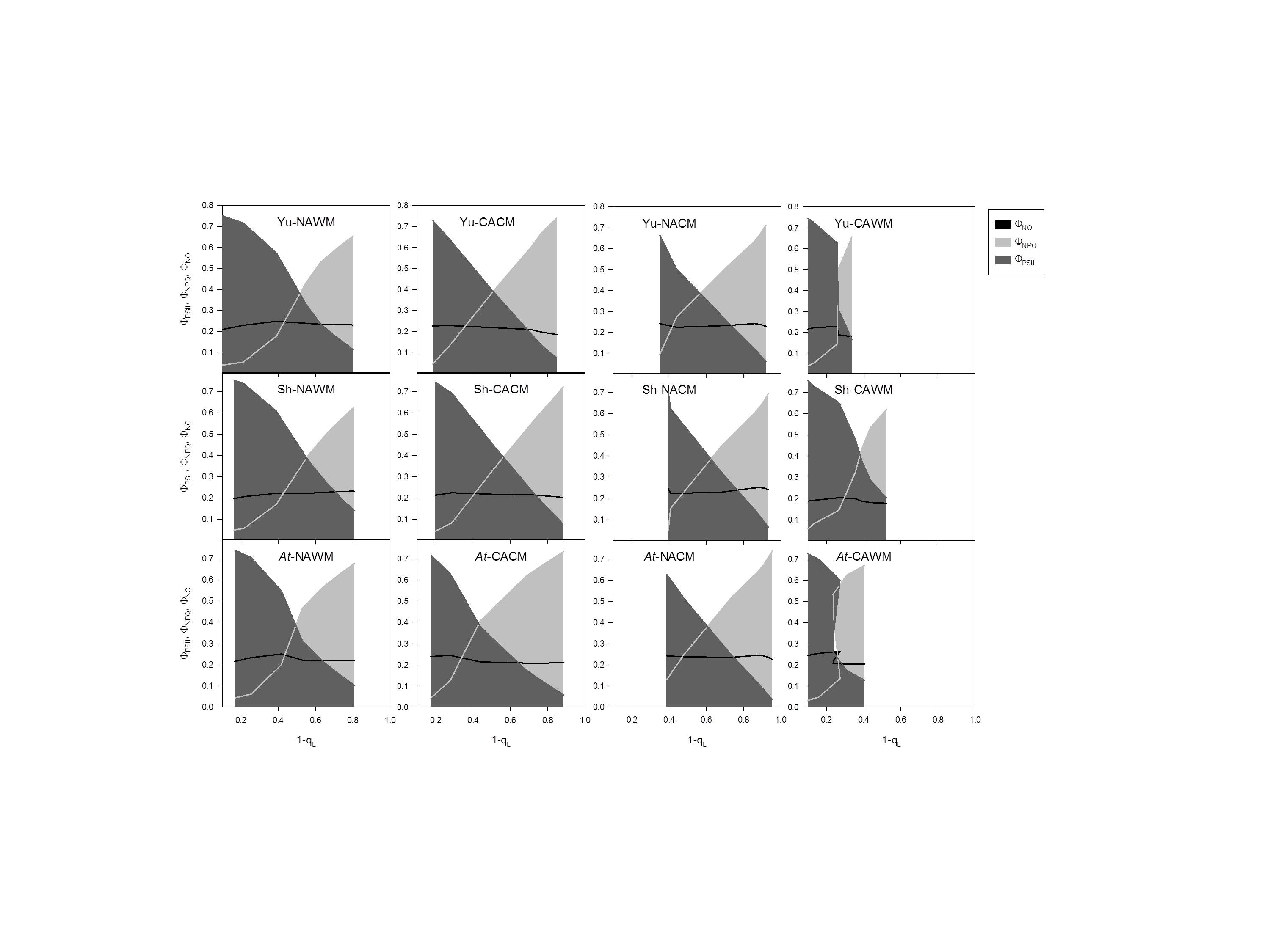

Supplement: Supplementary file 1 [file plants-06-00032-s001.zip › plants-206561-Supplementary Materials/Fig S3.JPG]

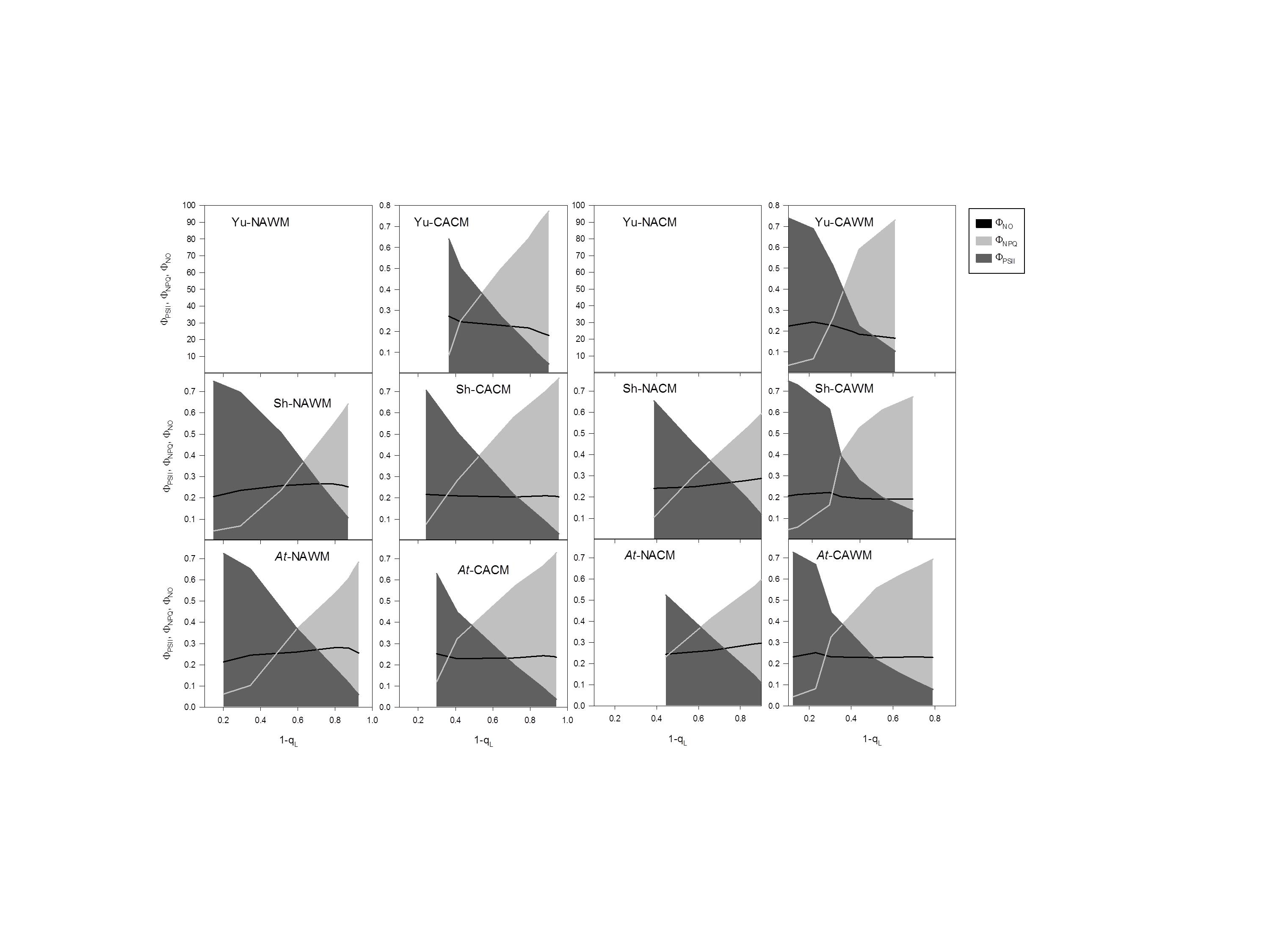

Supplement: Supplementary file 1 [file plants-06-00032-s001.zip › plants-206561-Supplementary Materials/Fig S4.JPG]
